# Eu(III) and Am(III) adsorption on aluminum (hydr)oxide minerals: surface complexation modeling

**DOI:** 10.1186/s12932-023-00081-5

**Published:** 2023-06-20

**Authors:** Anshuman Satpathy, Amy E. Hixon

**Affiliations:** Department of Civil and Environmental Engineering and Earth Sciences, University of Notre Dame, Notre Dame, IN 46556 USA

**Keywords:** Surface complexation modeling, Americium adsorption, Aluminum (hydr)oxide minerals, Corundum, Gamma-alumina, Gibbsite, Europium adsorption

## Abstract

Americium is a highly radioactive actinide element found in used nuclear fuel. Its adsorption on aluminum (hydr)oxide minerals is important to study for at least two reasons: (i) aluminum (hydr)oxide minerals are ubiquitous in the subsurface environment and (ii) bentonite clays, which are proposed engineered barriers for the geologic disposal of used nuclear fuel, have the same ≡AlOH sites as aluminum (hydr)oxide minerals. Surface complexation modeling is widely used to interpret the adsorption behavior of heavy metals on mineral surfaces. While americium sorption is understudied, multiple adsorption studies for europium, a chemical analog, are available. In this study we compiled data describing Eu(III) adsorption on three aluminum (hydr)oxide minerals—corundum (α-Al_2_O_3_), γ-alumina (γ-Al_2_O_3_) and gibbsite (γ-Al(OH)_3_)—and developed surface complexation models for Eu(III) adsorption on these minerals by employing diffuse double layer (DDL) and charge distribution multisite complexation (CD-MUSIC) electrostatic frameworks. We also developed surface complexation models for Am(III) adsorption on corundum (α-Al_2_O_3_) and γ-alumina (γ-Al_2_O_3_) by employing a limited number of Am(III) adsorption data sourced from literature. For corundum and γ-alumina, two different adsorbed Eu(III) species, one each for strong and weak sites, were found to be important regardless of which electrostatic framework was used. The formation constant of the weak site species was almost 10,000 times weaker than the formation constant for the corresponding strong site species. For gibbsite, two different adsorbed Eu(III) species formed on the single available site type and were important for the DDL model, whereas the best-fit CD-MUSIC model for Eu(III)-gibbsite system required only one Eu(III) surface species. The Am(III)-corundum model based on the CD-MUSIC framework had the same set of surface species as the Eu(III)-corundum model. However, the log K values of the surface reactions were different. The best-fit Am(III)-corundum model based on the DDL framework had only one site type. Both the CD-MUSIC and the DDL model developed for Am(III)-γ-alumina system only comprised of one site type and the formation constant of the corresponding surface species was ~ 500 times stronger and ~ 700 times weaker than the corresponding Eu(III) species on the weak and the strong sites, respectively. The CD-MUSIC model for corundum and both the DDL and the CD-MUSIC models for γ-alumina predicted the Am(III) adsorption data very well, whereas the DDL model for corundum overpredicted the Am(III) adsorption data. The root mean square of errors of the DDL and CD-MUSIC models developed in this study were smaller than those of two previously-published models describing Am(III)-γ-alumina system, indicating the better predictive capacity of our models. Overall, our results suggest that using Eu(III) as an analog for Am(III) is practical approach for predicting Am(III) adsorption onto well-characterized minerals.

**Graphical Abstract:**

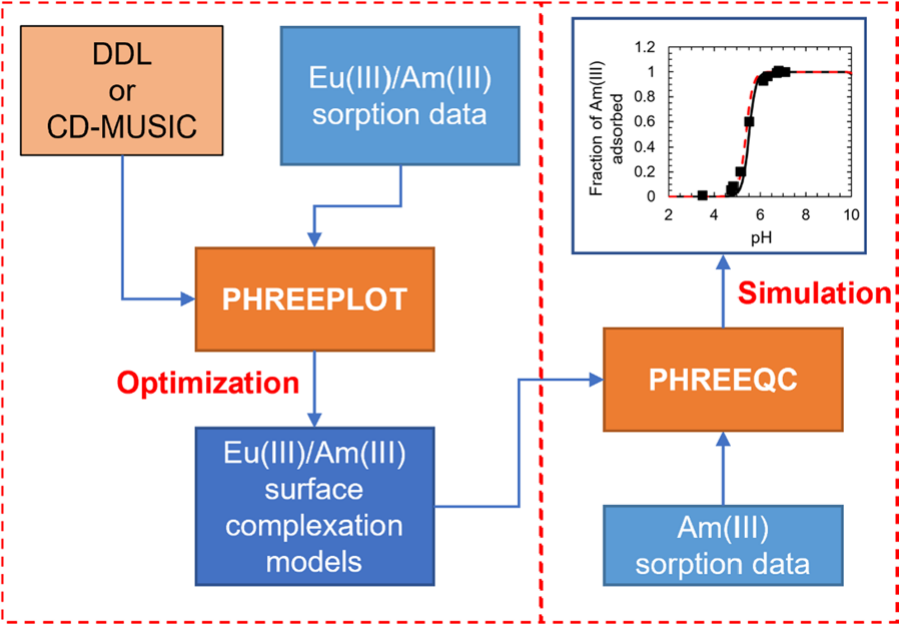

**Supplementary Information:**

The online version contains supplementary material available at 10.1186/s12932-023-00081-5.

## Introduction

Americium is a highly radioactive actinide element (e.g., t_1/2,Am-241_ = 432.6 a) that is formed in nuclear reactors and, thus, is present in used nuclear fuel as a minor actinide. Americium has also been introduced to the environment through nuclear weapons testing and aging legacy nuclear waste infrastructure. The emplacement of used nuclear fuel in deep underground repositories is being proposed as the best strategy for its long-term disposal [1–6]; commercially-available bentonite clay, which is mostly composed of the aluminum phyllosilicate mineral montmorillonite, will be used as a backfill material. Aluminum (hydr)oxide minerals like corundum (α-Al_2_O_3_), γ-alumina (γ-Al_2_O_3_), and gibbsite (γ-Al(OH)_3_) are ubiquitous in the subsurface environment and may influence the fate and transport of actinides (including americium). Furthermore, the aluminol sites present in aluminum (hydr)oxide minerals are also present in bentonite [7, 8]. Therefore, adsorption of americium on aluminum (hydr)oxide minerals is an important phenomenon to study.

Surface complexation modeling is a predictive tool that is often used to explain the adsorption of metal cations on minerals [9–12]. Surface complexes are analogous to aqueous complexes; however, unlike the aqueous complexation reaction, surface electrostatic effects, which are dependent on surface potential, need to be considered for the formation of surface complexes [10, 13–16]. Different types of electrostatic model frameworks can be used to develop surface complexation models for a given metal-mineral system and vary from one another in how they treat charge distribution at the mineral surface. Diffuse double layer (DDL) and charge distribution multi-site complexation (CD-MUSIC) are widely used electrostatic modeling frameworks for surface complexation modeling [9–11, 17]. In the CD-MUSIC framework, the charge on the mineral surface is distributed in three planes, which is a more realistic depiction of the charge distribution as compared to the DDL framework, where a point charge distribution is assumed. Although the CD-MUSIC framework is more realistic than the DDL framework, a larger number of parameters are required for defining the surface complexation modeling under the CD-MUSIC framework. These parameters are the inner and the outer layer capacitances and the charge distribution coefficients for the three planes (i.e., ΔZ_0_, ΔZ_1_, ΔZ_2_). The capacitances denote the rate of change of the surface potential as a function of distance from the surface. In the DDL framework, the surface potential remains constant between the naught plane and the d-plane and then decreases exponentially beyond the d-plane. Apart from the DDL and the CD-MUSIC framework, constant capacitance model (CCM) is also used for elucidating the surface electrostatics. In CCM, the surface potential decreases linearly from its maximum value on the naught plane to zero on the d-plane, and no surface effect is present beyond the d-plane. The details of different electrostatic models and their fundamentals have been explained in literature [18].

Europium is a lanthanide metal that resembles americium in its ionic size (1.066 Å and 1.09 Å for Eu and Am, respectively, in eightfold coordination), oxidation state, coordination number, and the properties of its coordination complexes [19, 20]. Therefore, europium is widely used as a chemical analog for americium. Surface complexation models have been developed for various metal ion sorption onto iron oxide minerals for a large and varied dataset sourced from multiple different studies which denote large variation in the input conditions [10, 21–25]. Many surface complexation models for Eu(III)-γ-alumina system have been developed since 2000 [26–28]. Rabung et al. [28] report the results of parameter optimization for Eu(III) adsorption to γ-alumina as a function of pH using a CCM electrostatic framework and two site types (strong and weak). The log K values for the same surface complex at different pH values varied, but were all close to 2.5; no global optimization was completed in order to obtain a unique log K for this system. Almost a decade later, two more surface complexation modeling studies [26, 27] were published for the Eu(III)-γ-alumina system. Kumar et al. [26] assume the presence of only a single surface site on γ-alumina and both monodentate and bidentate surface complexation of Eu(III) on the sorbent surface. The average log K values for the monodentate and the bidentate surface complexes are 2.22 ± 0.35 and − 4.99 ± 0.04, respectively. Although sorption edge data were collected as a function of Eu(III) concentration (0.1–100 µM), no global optimization was reported. Morel et al. [27] generate a surface complexation model for Eu(III) adsorption on γ-alumina based off a single set of adsorption edge data. They assume the presence of only one site and surface complex for optimization. The log K of the surface complex is − 1.2, which was almost six times weaker than the log K of the same surface complex optimized by Kumar et al. [26]. To the best of our knowledge, no surface complexation models have been developed for the Eu(III)-corundum and Eu(III)-gibbsite systems.

The objectives of this work are to compile all the available data for Eu(III) adsorption onto corundum, γ-alumina, and gibbsite, and Am(III) adsorption onto corundum and γ-alumina, use DDL and CD-MUSIC electrostatic frameworks to develop surface complexation models describing Eu(III) or Am(III) adsorption to these minerals, and determine whether the resulting Eu(III) surface complexation models could also describe the corresponding Am(III)-corundum and Am(III)-γ-alumina systems.

## Methods

### Aqueous speciation of Am(III) and Eu(III)

The aqueous speciation of both Am(III) and Eu(III) were determined for three different carbonate conditions: (i) no carbonate, (ii) carbonate in equilibrium with atmospheric CO_2_, and (iii) [CO_3_^2−^]_T_ = 10 mM. The set of aqueous complexation reactions, corresponding log K values, and specific ion interaction theory (SIT) coefficients of all the relevant cation–anion pairs were selected from the ThermoChimie database [29], as summarized in Additional file 1: Tables S1 and S2. Precipitation (e.g., through the formation of EuCO_3_OH_(cr)_) was allowed to occur.

### Selection of adsorption data

Data describing Eu(III) adsorption onto corundum (α-Al_2_O_3_), γ-alumina (γ-Al_2_O_3_), and gibbsite (γ-Al(OH)_3_) were sourced from the peer-reviewed literature and selected because the sorbent materials were well characterized [26–28, 30–32]. In total, 14 distinct datasets were compiled for the Eu(III)-corundum system (Additional file 1: Table S3), 15 distinct datasets were compiled for the Eu(III)-γ-alumina system (Additional file 1: Table S4), and 5 distinct datasets were compiled for the Eu(III)-gibbsite system (Additional file 1: Table S5); each dataset had a unique set of input parameters (i.e., total Eu(III) concentration, total mineral loading, carbonate condition, and ionic strength). For the Eu(III)-corundum and Eu(III)-γ-alumina systems, the specific surface area of the sorbent material varied between studies. For the Eu(III)-gibbsite system, all the sorption data were sourced from a single available study and, hence, only one gibbsite mineral with a unique specific surface area was employed. The total number of datapoints for the Eu(III)-corundum, Eu(III)-γ-alumina, and Eu(III)-gibbsite systems were 233, 160, and 77, respectively, and are available as Additional file 2.

A limited number of datasets describing Am(III) adsorption onto corundum (α-Al_2_O_3_) and γ-alumina (γ-Al_2_O_3_) were also sourced from the available literature [33–36]. In total, four different datasets each for corundum and gamma-alumina minerals were tabulated. Like europium data, each americium dataset comprised of a unique set of input parameters (i.e., total Am(III) concentration, total mineral loading, carbonate condition, and ionic strength). For the Am(III)-corundum system, the two different studies from which the adsorption data were sourced, used sorbent with different specific surface area. Whereas, for the Am(III)-gamma-alumina system, the sorbent used in all the datasets had the same specific surface area. The total number of datapoints for the Am(III)-corundum and Am(III)-γ-alumina systems were 26 and 46, respectively. These datapoints are also available as Additional file 2.

### Model development

**Fig. 1:**
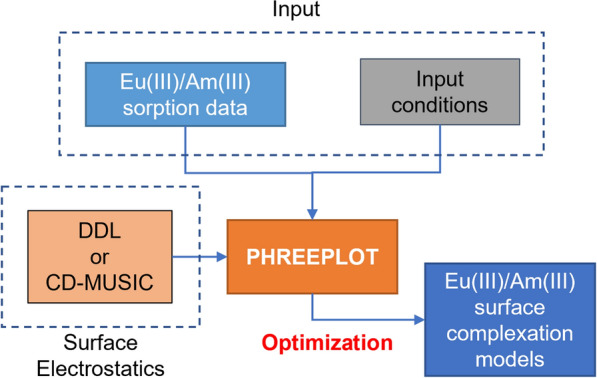
Surface complexation model development approach

The model development approach is illustrated in Fig. 1. Surface complexation models for the Eu(III)/Am(III)-corundum, Eu(III)/Am(III)-γ-alumina, and Eu(III)-gibbsite systems were developed using the DDL and CD-MUSIC electrostatic frameworks. The log K values for the surface protonation/deprotonation reactions for all three aluminum (oxy)hydroxide minerals were sourced from published potentiometric titration studies [37–40]. The DDL frameworks developed in this work were 2-pK_a_ models with a single set of protonation and deprotonation reactions for each mineral, whereas a 1-pK_a_ model was used for the CD-MUSIC frameworks. Apart from protonation, outer sphere complexation with Na^+^ and Cl^−^/ClO_4_^−^ and inner sphere complexation with carbonate were also assumed in the CD-MUSIC models. The log K values for these reactions were adopted from previous studies [37–39, 41]. Tables 1 and 2 include the protonation/deprotonation and background ion surface complexation reactions and log K values selected in this work.

**Table 1:** DDL models for Eu(III)/Am(III) adsorption on aluminum (hydr)oxide minerals

Surface complexation reaction	log K		
	Corundum	γ-Alumina	Gibbsite
$\equiv AlOH+{H}^{+}\leftrightarrow { \equiv AlOH}_{2}^{+}$	6.03 ± 0.25^a^	8.50 ± 0.29^a^	6.78 ± 0.29^a^
$\equiv AlOH\leftrightarrow \equiv Al{O}^{-}+{H}^{+}$	− 7.47 ± 0.42^a^	− 9.20 ± 0.52^a^	− 10.10 ± 0.43^a^
$\equiv {Al}_{s}OH+{Eu}^{+3}\leftrightarrow \equiv {Al}_{s}O{Eu}^{+2}+{H}^{+}$	N/A	N/A	N/A
$\equiv {Al}_{s}OH+{Eu}^{+3}+{H}_{2}O\leftrightarrow \equiv {Al}_{s}O{EuOH}^{+}+{2H}^{+}$	− **1.54 ± 0.16**	**0.50 ± 0.08**	N/A
$\equiv {Al}_{w}OH+{Eu}^{+3}\leftrightarrow \equiv {Al}_{w}O{Eu}^{+2}+{H}^{+}$	N/A	N/A	**3.25 ± 0.22**
$\equiv {Al}_{w}OH+{Eu}^{+3}+{H}_{2}O\leftrightarrow \equiv {Al}_{w}O{EuOH}^{+}+{2H}^{+}$	− **5.45 ± 0.18**	− **5.03 ± 0.08**	− **5.10 ± 0.23**
*RMSE*	0.1978	0.1166	0.1799
*R*^2^	0.7804	0.9229	0.8282
*Correlation coefficient*	0.146	− 0.023	− 0.124
$\equiv AlOH+{Am}^{+3}+{H}_{2}O\leftrightarrow \equiv AlO{AmOH}^{+}+{2H}^{+}$	− **3.91 ± 0.29**	− **2.36 ± 0.08**	N/A
*RMSE*	0.1907	0.1059	N/A
*R*^2^	0.7856	0.9323	N/A

Optimized values of the fit parameters are provided in bold text

^a^Yang et al. [40]

**Table 2:** CD-MUSIC models for Eu(III)/Am(III) adsorption on aluminum (hydr)oxide minerals

Surface complexation reaction	ΔZ_0_	ΔZ_1_	ΔZ_2_	log K		
				Corundum	γ-Alumina	Gibbsite
${\equiv AlOH}^{-0.5}+{H}^{+}\leftrightarrow { \equiv AlOH}_{2}^{+0.5}$	1	0	0	9.6^a^	9.0^b^	9.87^c^
${\equiv AlOH}^{-0.5}+{Na}^{+}\leftrightarrow {\equiv AlOH}^{-0.5}{Na}^{+}$	–	–	–	− 0.8^a^	− 0.096^b^	0.7^c^
${\equiv AlOH}^{-0.5}+{H}^{+}+ {Cl}^{-}\leftrightarrow { \equiv AlOH}_{2}^{+0.5}{Cl}^{-}$	–	–	–	8.00^a^	8.82^b^	9.74^c^
${\equiv AlOH}^{-0.5}+{H}^{+}+ {C{O}_{3}}^{-2}\leftrightarrow {\equiv AlOCOO}^{-1.5}+{H}_{2}O$	− 1	0	0	− 0.3^d^	− 0.3^d^	− 0.3^d^
${\equiv {Al}_{s}OH}^{-0.5}+{Eu}^{+3}+{H}_{2}O\leftrightarrow { \equiv {Al}_{s}OEuOH}^{+0.5}+2{H}^{+}$	0.5	0.5	0	**1.61 ± 0.12**	**0.89 ± 0.08**	N/A
${\equiv {Al}_{w}OH}^{-0.5}+{Eu}^{+3}+{H}_{2}O\leftrightarrow { \equiv {Al}_{w}OEuOH}^{+0.5}+2{H}^{+}$	0.5	0.5	0	− **2.71 ± 0.09**	− **4.63 ± 0.07**	− **2.99 ± 0.24**
*RMSE*				0.1766	0.1134	0.1982
*R*^*2*^				0.8235	0.9261	0.7914
*Correlation coefficient*				− 0.111	− 0.009	N/A
${\equiv {Al}_{s}OH}^{-0.5}+{Am}^{+3}+{H}_{2}O\leftrightarrow { \equiv {Al}_{s}OAmOH}^{+0.5}+2{H}^{+}$	0.5	0.5	0	**3.45 ± 2.72**	N/A	N/A
${\equiv {Al}_{w}OH}^{-0.5}+{Am}^{+3}+{H}_{2}O\leftrightarrow { \equiv {Al}_{w}OAmOH}^{+0.5}+2{H}^{+}$	0.5	0.5	0	− **1.47 ± 0.27**	− **1.93 ± 0.08**^**#**^	N/A
*RMSE*				0.1818	0.1001	N/A
*R*^*2*^				0.8129	0.9382	N/A
*Correlation coefficient*				− 0.044	N/A	N/A

Optimized values of the fit parameters are provided in bold text

^#^Only one site type was assumed for model optimization

^a^Janot et al. [37]

^b^Mayordomo et al. [38]

^c^Weerasooriya et al. [39]

^d^Wijnja and Schulthess [41]

The PHREEQC-based optimization tool, PhreePlot, was used for parameter optimization. For corundum and γ-alumina, two site types—strong and weak—were chosen. The fraction of strong sites required for a good fit, based on a comparison of root mean square of errors (RMSE), were found to be 0.05% and 0.1% for corundum and γ-alumina, respectively. For gibbsite, only one site type was considered since no improvement in fitting quality was observed after initial optimization attempts with two site types. A site density of 2.31 sites·nm^−2^ was employed in all systems [9]. The Eu(III)-aluminum (hydr)oxide surface complexation reactions were considered based on the dominant aqueous Eu(III) species at the pH range of interest (Tables 1 and 2). For the corundum and γ-alumina systems, only one Eu(III) surface complex was important for each site type. For the gibbsite system, two surface complexes for the only surface site type were considered (Tables 1 and 2). The models were optimized for a minimum residual sum of squares between the observed and the model generated values of the fraction of europium adsorbed (Additional file 3).

The Am(III) surface complexation models on corundum and γ-alumina adopted the same sorbent parameters as those for the Eu(III) surface complexation models. However, as there was no variation in the total Am(III) concentration in the Am(III)-γ-alumina data, only one site type was adopted for optimization of the surface complexation model for Am(III)-γ-alumina system. In the case of Am(III)-corundum system, the DDL model with two site types resulted in no better fit than the one with one site type. Hence, only a one site type DDL model was adopted for comparison. The Am(III) surface complexation reactions for both the Am(III)-corundum and the Am(III)-γ-alumina systems were analogous to the corresponding Eu(III) surface complexation reactions (Tables 1 and 2).

## Results and discussion

### Aqueous speciation of Am(III) and Eu(III)

The aqueous speciation trends for Am(III) were found to be similar to those of Eu(III) for the three different carbonate conditions we examined in this work: (i) no carbonate, (ii) carbonate in equilibrium with atmospheric CO_2_, and (iii) [CO_3_^2−^]_T_ = 10 mM (see Additional file 1: Figure S1). In the absence of carbonate, Eu^3^/Am^3+^ and EuCl^+2^/AmCl^+2^ were the most stable aqueous species at pH < 7, whereas hydrolysis species (e.g., EuOH^2+^, Eu(OH)_2_^+^, and Eu(OH)_3_(aq)) dominated the aqueous speciation at pH > 7. For systems ii and iii, which contained dissolved inorganic carbon (DIC), Eu^+3^/Am^+3^ and EuCl^+2^/AmCl^+2^ were still the most dominant species at low pH (< 6), but both americium and europium formed strong aqueous carbonate complexes (e.g., EuCO_3_^+^, Eu(CO_3_)_2_^−^ Eu(CO_3_)_3_^3−^) at pH > 6. For systems in equilibrium with atmospheric CO_2_, the Am(III)/Eu(III)-carbonate complexes remained dominant for the entire pH range above 6. For the closed system with a fixed amount of DIC (10 mM), the Am(III)/Eu(III)-carbonate complexes remained dominant only in the near-neutral to alkaline pH range (~ 6 to ~ 11). At a highly alkaline pH (> 11), Am(III)/Eu(III)-hydroxyl complexes (e.g., Eu(OH)_2_^+^ and Eu(OH)_3_) were the dominant species.

### Surface complexation models describing Eu(III) adsorption to corundum

The surface complexation models developed for Eu(III)-corundum system performed well for the large range of datasets that were used for model development (see Tables 1 and 2). The overall root mean square of errors (RMSEs) were 0.1978 and 0.1766 for the DDL and the CD-MUSIC electrostatic frameworks, respectively. This indicates overall better performance of the CD-MUSIC model as compared to the DDL model. However, for different individual datasets, the relative performance of the two different types of models varied (Additional file 1: Figure S7).

**Fig. 2:**
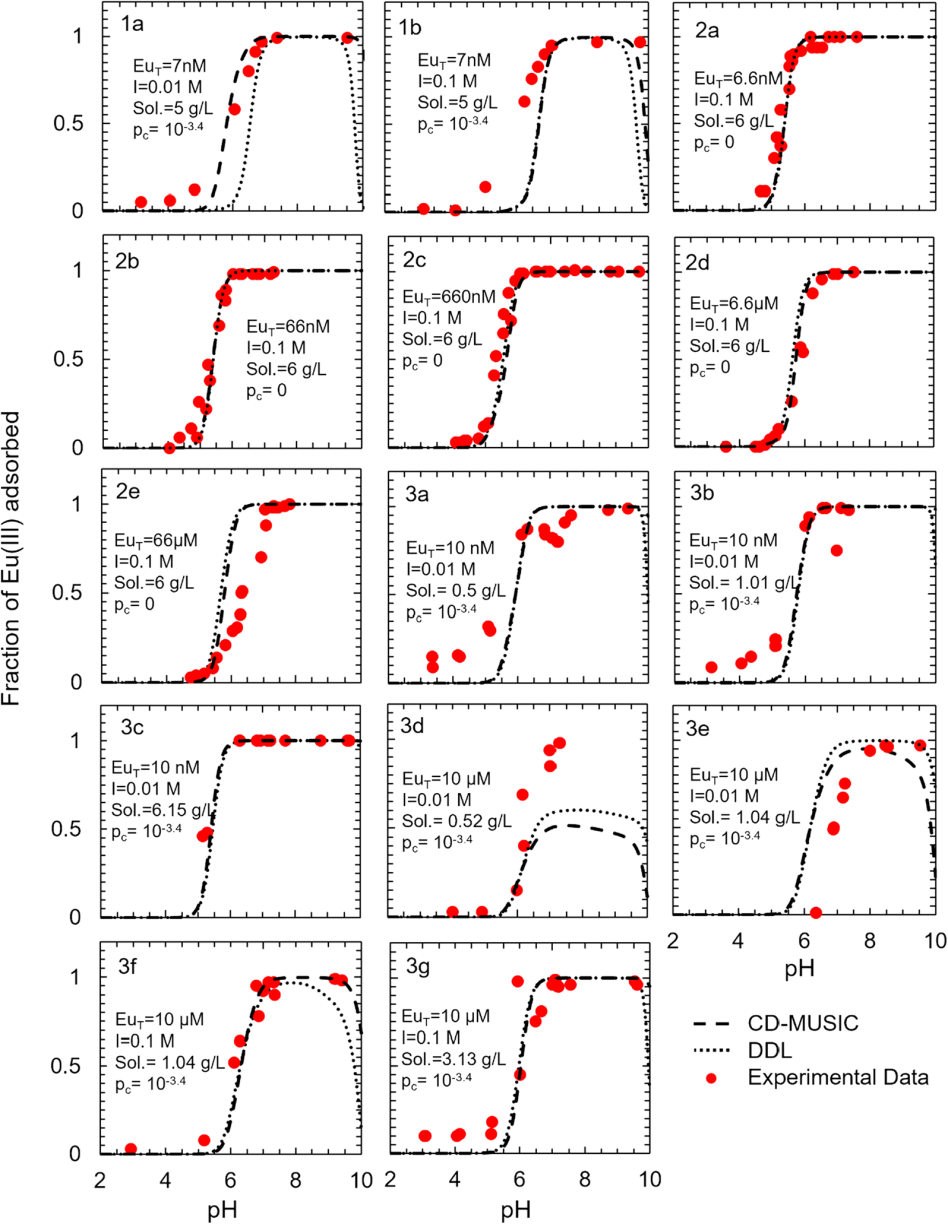
Surface complexation models describing Eu(III) adsorption to corundum as a function of total europium concentration (Eu_T_), ionic strength (I), sorbent solid concentration (Sol.), and partial pressure of CO_2(g)_ (p_c_). Experimental data were derived from (1) Norden et al. [32] (2) Kupcik et al. [31] and (3) Baumer et al. [30] and the full experimental conditions are summarized in Additional file 1: Table S3. The contributions of each individual surface species are illustrated in Additional file 1: Figures S2 (DDL) and S3 (CD-MUSIC)

The DDL model predictions were either close to or below the observed experimental values for most input conditions (Fig. 2). The only exceptions were input conditions 2e (66 µM total Eu(III), 0.1 M ionic strength, 6 g L^−1^ corundum, no carbonate) and 3e (10 µM total Eu(III), 0.01 M ionic strength, 1.04 g L^−1^ corundum, 10^–3.4^ atm CO_2(g)_), where the DDL model overpredicted Eu(III) adsorption. This could be because of the overestimation of the adsorption sites. On the other hand, the CD-MUSIC model did not overpredict Eu(III) adsorption by a large margin for any input condition. The estimation of the adsorption sites could be done better in the CD-MUSIC framework as compared to the DDL framework because the effect of the counter cation on the surface charge was also accounted in the CD-MUSIC framework. The adsorption predictions of both the DDL and the CD-MUSIC models for input condition 3d (10 μM total Eu(III), 0.01 M ionic strength, 0.52 g L^−1^ corundum, 10^–3.4^ atm CO_2(g)_) result from fewer sorption sites being available relative to the total Eu(III) concentration.

### Surface complexation models describing Eu(III) adsorption to γ-alumina

The overall RMSEs of the DDL and the CD-MUSIC models for Eu(III)-γ-alumina systems developed in this study (Tables 1 and 2) were 0.1166 and 0.1134, respectively, indicating only marginally better performance of the CD-MUSIC model as compared to the DDL model. The relative performance of the DDL and the CD-MUSIC models for the individual datasets did not show much variation (Additional file 1: Figure S7).

**Fig. 3:**
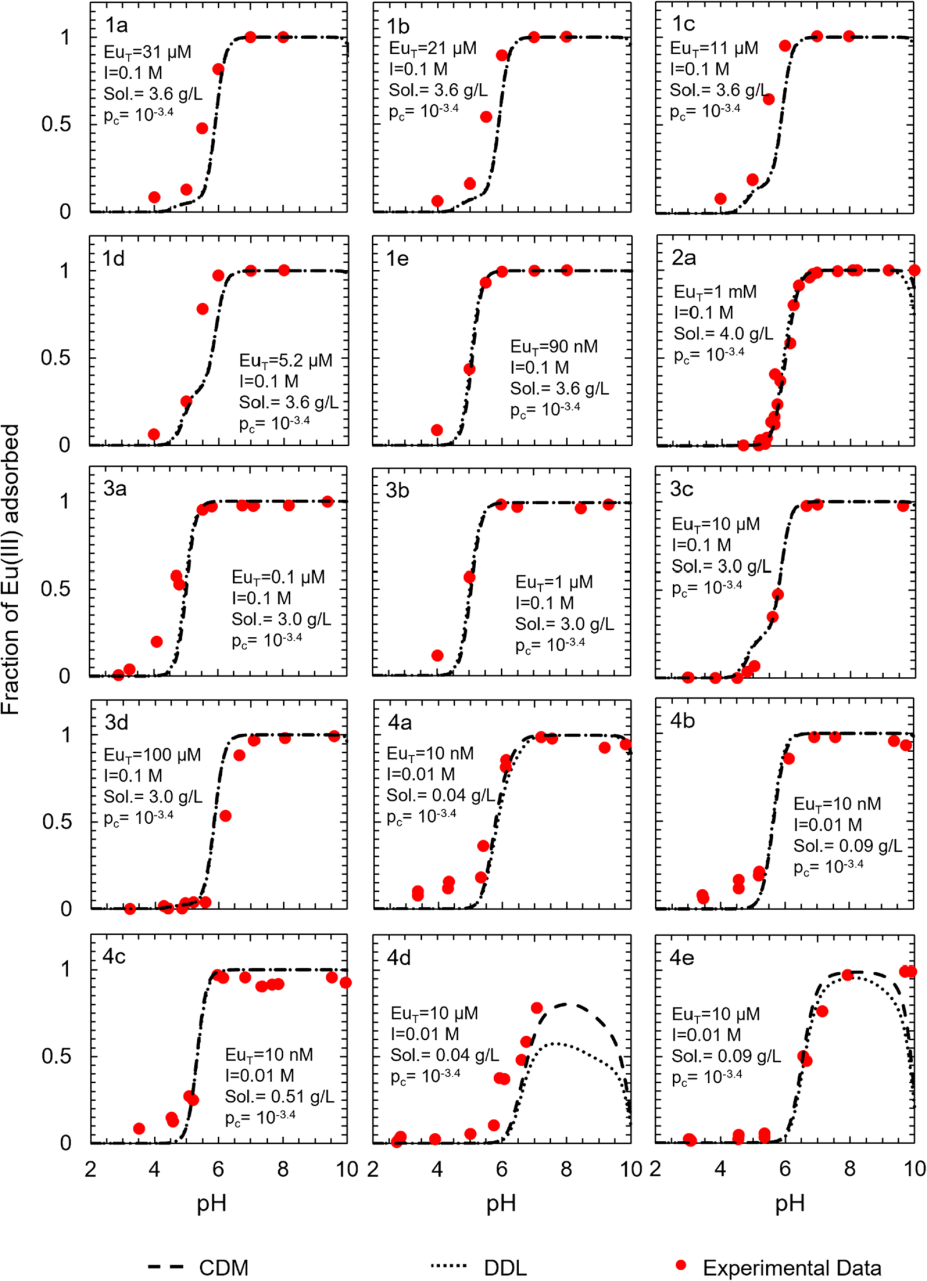
Surface complexation models describing Eu(III) adsorption to gamma-alumina as a function of total europium concentration (Eu_T_), ionic strength (I), sorbent solid concentration (Sol.), and partial pressure of CO_2(g)_ (p_c_). Experimental data were derived from (1) Rabung et al. [28] (2) Morel et al. [27] (3) Kumar et al. [42] and (4) Baumer et al. [30] and full experimental conditions are summarized in Additional file 1: Table S4. The contributions of each individual surface species are illustrated in Additional file 1: Figures S4 (DDL) and S5 (CD-MUSIC)

Like the Eu(III)-corundum system, no adsorption was predicted for pH < 4 and adsorption increased to ~ 100% between pH 4 and 7 and the adsorption edge shifted to higher pH with increasing Eu_T_ (Fig. 3). However, unlike the Eu(III)-corundum system, no prominent carbonate effect was observed above pH 7. This is because, at a given pH, the adsorption affinity of the positively charged Eu(III) species will be higher on γ-alumina as compared to that on corundum due to the higher pH_pzc_ of γ-alumina as compared to that of corundum. Neither electrostatic model highly overpredicted adsorption for any input conditions over any pH range. This shows that both the electrostatic frameworks were equally helpful in developing a more robust surface complexation model for Eu(III)-γ-alumina system.

### Surface complexation models describing Eu(III) adsorption to gibbsite

The overall RMSEs of the DDL and the CD-MUSIC models for Eu(III)-gibbsite system developed in this study (Tables 1 and 2) were 0.1799 and 0.1982, respectively, indicating better performance of the DDL model as compared to the CD-MUSIC model. The variation of RMSEs for each individual dataset can be seen in Additional file 1: Figure S7.

**Fig. 4:**
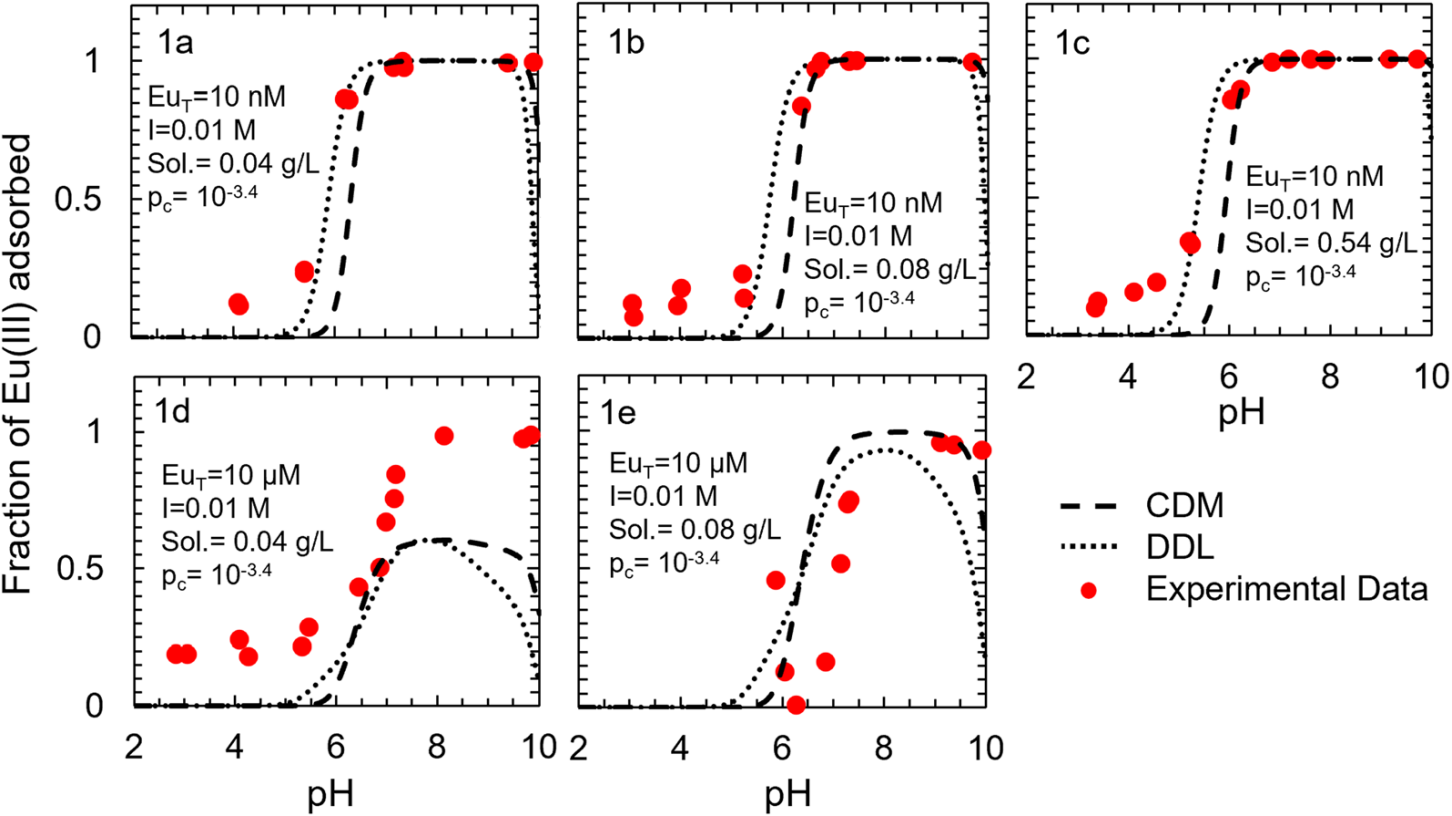
Surface complexation models describing Eu(III) adsorption to gibbsite as a function of total europium concentration (Eu_T_), ionic strength (I), sorbent solid concentration (Sol.), and partial pressure of CO_2(g)_ (p_c_). Experimental data were derived from Baumer et al. [30] and full experimental conditions are summarized in Additional file 1: Table S5. The contributions of each individual surface species for the DDL model are illustrated in Additional file 1: Figure S6

Unlike the datasets employed in model development for the Eu(III)-corundum and the Eu(III)-γ-alumina systems, the datasets used in developing the models for the Eu(III)-gibbsite system were sourced from one single study. As with the Eu(III)-corundum and Eu(III)-γ-alumina systems, the adsorption edge is observed between pH 4 and 7 for most conditions (Fig. 4). The only exception is condition 1d (10 μM total Eu(III), 0.01 M ionic strength, 0.04 g L^−1^ gibbsite, 10^–3.4^ atm CO_2(g)_), where the highest predicted adsorption is only ~ 50%. This could be because of the low sorption site concentration relative to the total Eu(III) concentration. For pH < 6, both models underpredicted Eu(III) adsorption to gibbsite, indicating the presence of an impurity in the solid phase (e.g., clays that contribute cation exchange capacity) or alternate sorption mechanism that is not accounted for in the models. For pH > 6, the adsorption predictions for both the DDL and the CD-MUSIC models varied according to the total Eu(III) concentration. For a low total Eu(III) concentration (~ 10 nM), adsorption predictions were close to the observed values. However, no good adsorption predictions were observed for a high total Eu(III) concentration.

### Surface complexation models describing Am(III) adsorption to corundum

The overall RMSEs of the DDL and the CD-MUSIC models developed using the limited Am(III)-corundum adsorption data were 0.1907 and 0.1818, respectively, indicating slightly better performance of the CD-MUSIC model as compared to the DDL model. RMSEs of the models for each individual dataset are shown in Additional file 1: Figure S8.

**Fig. 5:**
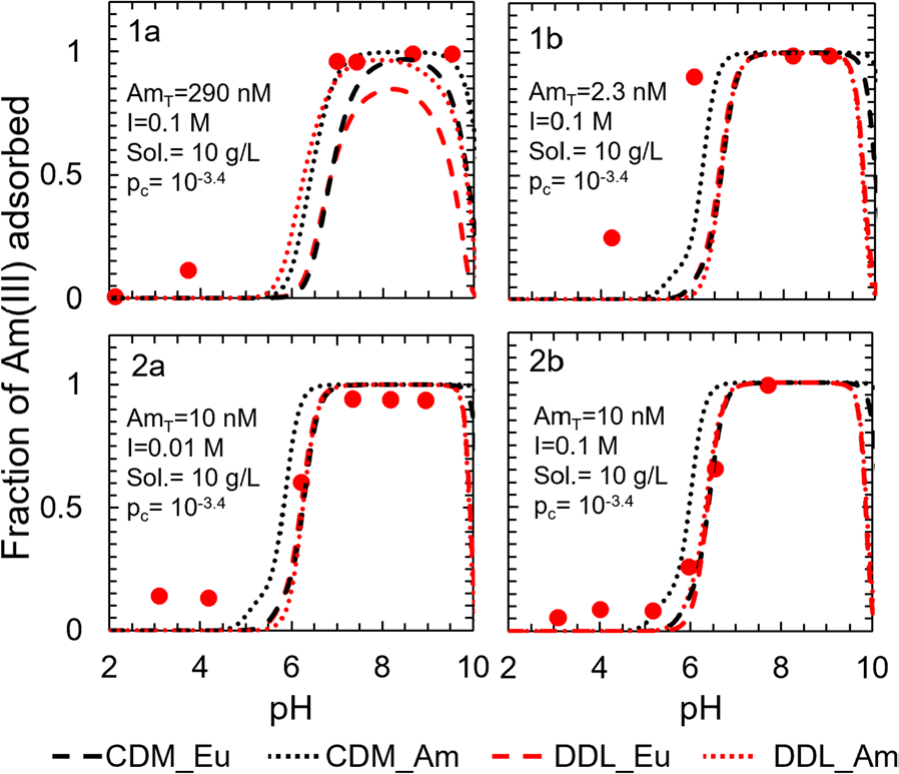
Surface complexation models describing Am(III) adsorption to corundum as a function of total americium concentration (Am_T_), ionic strength (I), sorbent solid concentration (Sol.), and partial pressure of CO_2(g)_ (p_c_). The CDM_Eu curves represent a CD-MUSIC model developed by employing europium sorption data, the CDM_Am curves represent a CD-MUSIC model developed by employing americium sorption data, the DDL_Eu curves represent a DDL model developed by employing europium sorption data, and the DDL_Am curves represent a DDL model developed by employing americium sorption data. Experimental data were derived from (1) Allard et al. [33] and (2) Moulin et al. [34] and full experimental conditions are summarized in Additional file 1: Table S6. The contributions of each individual surface species are illustrated in Additional file 1: Figures S9 (CDM_Eu), S10 (CDM_Am), S11 (DDL_Eu) and S12 (DDL_Am)

Like the Eu(III)-corundum system, the adsorption edge was located between pH 4 and pH 6 for most input conditions (Fig. 5). The adsorption prediction of the DDL model was lower than that of the CD-MUSIC model for the acidic pH range (< 7) and for pH > 9, whereas between pH 7 and 9, the sorption prediction of both the models were close to each other. Both models underpredict Am(III) sorption at pH < 6, but satisfactorily predict the experimental data under near-neutral and basic conditions (Fig. 5).

### Surface complexation models describing Am(III) adsorption to γ-alumina

The overall RMSEs of the DDL and CD-MUSIC models developed using the limited Am(III)-γ-alumina adsorption data were 0.1059 and 0.1001, respectively, indicating almost equal performance of the CD-MUSIC and the DDL models. RMSEs of the models for each individual dataset are provided in Additional file 1: Figure S8.

**Fig. 6:**
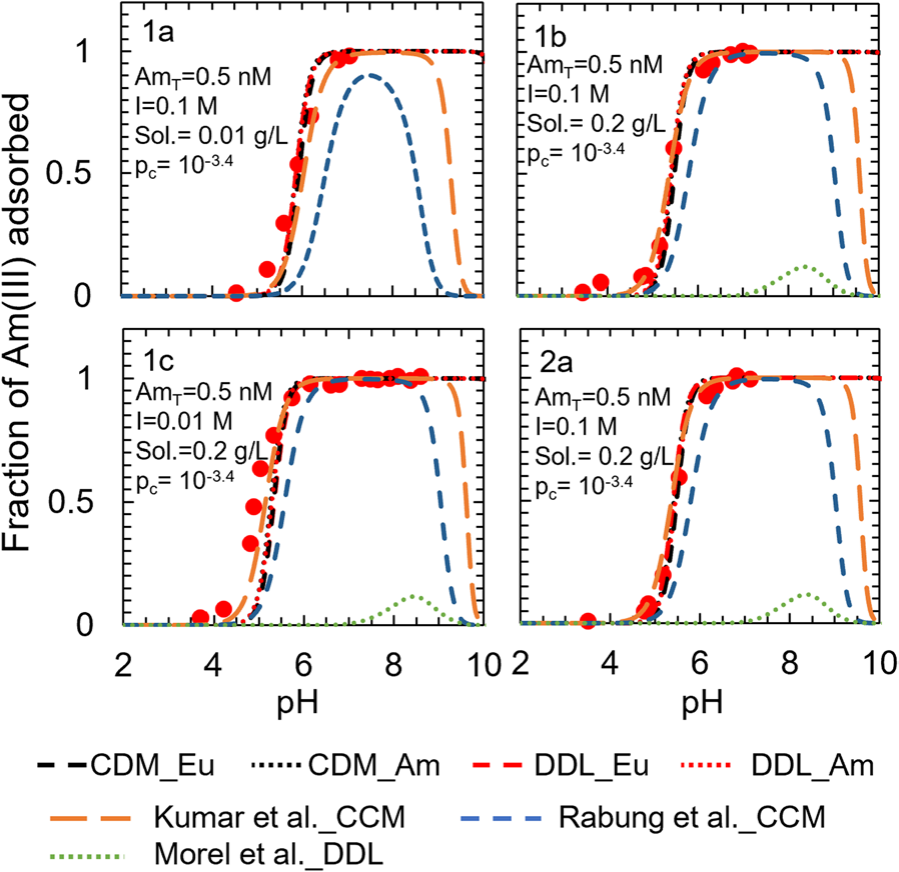
Surface complexation models describing Am(III) adsorption to γ-alumina. Experimental conditions from (1) Righetto et al. [36] and (2) Righetto et al. [35] and full experimental conditions are summarized in Additional file 1: Table S7. The contributions of each individual surface species are illustrated in Additional file 1: Figures S13 (CDM_Eu) and S14 (DDL_Eu). The CDM_Eu curve represents a CD-MUSIC model developed by employing europium sorption data, the CDM_Am curve represents a CD-MUSIC model developed by employing americium sorption data, the DDL_Eu curve represents a DDL model developed by employing europium sorption data, and the DDL_Am curve represents a DDL model developed by employing americium sorption data. The curves labeled Kumar et al. _CCM, Rabung et al. _CCM and Morel et al. _DDL are previous models sourced from literature [26–28]

The adsorption prediction of both the DDL and the CD-MUSIC models were close to the observed adsorption for most input conditions for the entire pH range of 2 to 12 (Fig. 6). The only exception was dataset 1c (0.5 nM total Am(III), 0.01 M ionic strength, 0.2 g L^−1^ γ-alumina, 10^–3.4^ atm CO_2(g)_), for which both the DDL and the CD-MUSIC models underpredicted adsorption at pH ≤ 5.

### Performance and validation of the surface complexation models

We were interested in knowing whether the models developed to describe Eu(III) adsorption to aluminum (hydr)oxide minerals could also be used to describe Am(III) adsorption, and how do they perform as compared to the models developed by employing Am(III) adsorption data. Model simulations (Fig. 5) were generated for the experimental conditions described in Allard et al. [33] and Moulin et al. [34] for americium adsorption to corundum using the log K values in Tables 1 and 2. All four models underpredicted adsorption at pH < 5, which may suggest the presence of an impurity or alternate sorption mechanism that is not accounted for in the models. Over the pH range 5 to 7, the CD-MUSIC model developed using the Am(III) data predicted a higher fraction of sorbed americium than the CD-MUSIC model developed using the Eu(III) data, whereas the predictions from the DDL models varied according to the total Am(III) concentration in the system. When the total americium concentration was ≤ 10 nM, adsorption predictions of the DDL model developed using Am(III) data were same as those of the DDL model developed using the Eu(III) data. At higher Am(III) concentration (i.e., 290 nM in dataset 1a), adsorption predictions of the DDL model developed using the Am(III) data were much higher than those of the DDL model developed using the Eu(III) data. This can be attributed to the absence of two site types in the DDL model optimized using the Am(III) data, which would have accounted for the variation in the Am(III) concentration. The variation in the RMSEs of each model for the individual datasets is shown in Additional file 1: Figure S8. Although the surface complexation reaction constants optimized using the Eu(III) and the Am(III) sorption data differ from each other (Tables 1 and 2), the simulations generated by the Eu(III) model and the Am(III) model for the Am(III) sorption data were similar. This result indicates that the currently-available Am(III) sorption data can be predicted through multiple combinations of surface complexation reaction constants that can mathematically constrain the chemical equilibrium problem.

The validation of the models for the γ-alumina system were performed by generating adsorption edge simulations for the experimental conditions described in Righetto et al. [36]. Both the DDL and the CD-MUSIC models developed in this study predict Am(III) adsorption onto γ-alumina closely and represent a better fit to the experimental data than some other published models [27, 28] (Fig. 6). Unlike the models developed in this study, the published models sourced from Rabung et al. [28] and Morel et al. [27] (Additional file 1: Table S6) were developed by employing Eu(III) adsorption data sourced from a single study. Both of these published models underpredict Am(III) adsorption to γ-alumina for the entire pH range of 2–12, with the Morel et al. [27] model predictions being significantly lower than the experimental data for all the datasets. The latter results from an equilibrium constant (log K_1_ = − 1.2) for the $\equiv AlOH+{Am}^{+3}\leftrightarrow {\equiv AlOAm}^{+2}+{H}^{+}$ reaction that is almost 3000 times weaker than the equilibrium constant of the corresponding surface complexation reaction in the Rabung et al. [27] models (log K_1_ = 2.21). The adsorption predictions for the model sources from Kumar et al. [26] were close to the observed data for all the four datasets, and its performance was as good as the models developed in the present work (see Additional file 1: Figure S8). The model developed in Kumar et al. [26] included a bidentate surface complexation reaction ($\equiv AlOH+{Am}^{+3}\leftrightarrow \left({{\equiv AlO)}_{2}Am}^{+}+{2H}^{+}\right).$ Conversely, the models developed in the present work considered only monodentate complexation for both Eu(III) and Am(III) on alumina mineral surfaces, which is consistent with the X-ray absorption fine structure spectroscopy of Eu(III) adsorption onto γ-alumina, where no evidence for formation of any bidentate surface species of Eu(III) was found [43].

## Conclusions

Surface complexation models were developed using data from the literature describing Eu(III) adsorption to three different aluminum (hydr)oxide minerals—corundum, γ-alumina, and gibbsite—and applying diffuse double layer (DDL) and charge distributed multisite complexation (CD-MUSIC) electrostatic frameworks. The DDL and CD-MUSIC models showed varying degrees of effectiveness in describing Eu(III) adsorption to corundum and gibbsite. However, both models predicted Eu(III) adsorption to γ-alumina very well. The choice of modeling framework was not sensitive to the model performance. Surface complexation models describing adsorption of Am(III) to corundum and γ-alumina were also generated by employing a limited number of Am(III) adsorption data available in literature. The models developed for Eu(III) adsorption to corundum and γ-alumina were employed for generating adsorption simulations describing Am(III) adsorption to these minerals, and their performances were compared with the models developed by employing Am(III) adsorption data [26, 28, 44]. For most Am(III) adsorption datasets, the performance of the models developed by employing Eu(III) adsorption data was as good as the models developed by employing Am(III) adsorption data. The Am(III) adsorption predictions of all four models developed for corundum were below the observed data at low pH. Whereas, the Am(III) adsorption to γ-alumina was predicted very well by all four models developed in this study. Although the aqueous chemistry of Eu(III) and Am(III) are similar, the surface complexation reaction constants optimized for the Eu(III) and Am(III) sorption data were noticeably different. This could be attributed to the lack of variation in the input conditions employed for Am(III) sorption data as compared to those for Eu(III) sorption data. Whereas the Eu(III) sorption data were sourced from studies which reflect several orders of magnitude variation in total sorbate concentration (i.e., from 6.6 nM to 10 µM for corundum, and 10 nM to 100 µM for γ-alumina), the Am(III) sorption data represent a much smaller range of sorbent concentrations (i.e., 2.3–290 nM for corundum and 0.5 nM for γ-alumina). Thus, the optimization of Am(III) sorption is biased towards the less varied total Am(III) concentration in the input conditions. The performance of the models developed for Am(III)-γ-alumina system were compared with three models available in literature. Our models performed better than two out of the three previous models when predicting Am(III) adsorption on γ-alumina. The adsorption predictions of one of the previous models (i.e., Kumar et al. [26]), was at par with the models developed in this work. However, unlike the Kumar et al. [26] model, our models assumed only monodentate surface speciation of americium, which is consistent with published X-ray fine structure spectroscopy analyses [43]. This work helps us better predict the adsorption trends of Am(III) onto common alumina (hydr)oxide minerals under varied input conditions, and thus, towards a better understanding of the fate and transport of Am(III) in subsurface environment.

## Supplementary Information

**Additional file 1. ** Supporting Information

**Additional file 2. ** Sorption Database

**Additional file 3. ** Example Optimization File

## Data Availability

The datasets supporting the conclusions of this article are included within the article (and its additional files).

## Abbreviations

CCM: Constant capacitance model
CD-MUSIC: Charge distribution multisite complexation
DDL: Diffuse double layer
NEA-TDB: Nuclear Energy Agency Thermochemical Database
RMSE: Root mean square of errors
SIT: Specific ion interaction theory
