# Supplementary material for: Eu(III) and Am(III) adsorption on aluminum (hydr)oxide minerals: surface complexation modeling
Source: Geochem Trans. 2023 Jun 20;24:2. doi: 10.1186/s12932-023-00081-5 (PMC10283230; doi:10.1186/s12932-023-00081-5)
Supplement: Supplementary file 1 — Additional file 1. Supporting Information [file 12932_2023_81_MOESM1_ESM.pdf]

Eu(III) and Am(III) adsorption on aluminum (hydr)oxide minerals: Surface complexation modeling

Anshuman Satpathy and Amy E. Hixon*

Department of Civil and Environmental Engineering and Earth Sciences, University of Notre Dame, Notre Dame, Indiana 46556 USA

*Corresponding Author: ahixon@nd.edu

**Content**	**Page Number**
Table S1	2
Table S2	2
Table S3	4
Table S4	5
Table S5	5
Table S6	7
Figure S1	3
Figure S2	6

Table S1. Aqueous complexation reactions for Eu(III) and Am(III) with equilibrium constants.

Reactions	log K
$Eu^{+3} + H_2O \leftrightarrow EuOH^{+2} + H^+$	-7.8
$Eu^{+3} + 2H_2O \leftrightarrow Eu(OH)_2{}^+ + 2H^+$	-15.7
$Eu^{+3} + 3H_2O \leftrightarrow Eu(OH)_3 + 3H^+$	-26.2
$Eu^{+3} + 4H_2O \leftrightarrow Eu(OH)_4{}^- + 4H^+$	-40.7
$Eu^{+3} + CO_3^{-2} \leftrightarrow EuCO_3{}^+$	7.9
$Eu^{+3} + 2CO_3^{-2} \leftrightarrow Eu(CO_3)_2{}^-$	12.9
$Eu^{+3} + 3CO_3^{-2} \leftrightarrow Eu(CO_3)_3{}^{-3}$	14.8
$Eu^{+3} + H^+ + CO_3^{-2} \leftrightarrow EuHCO_3{}^{+2}$	12.43
$Eu^{+3} + Cl^- \leftrightarrow EuCl^{+2}$	0.76
$Eu^{+3} + 2Cl^- \leftrightarrow EuCl_2{}^+$	-0.05
$Am^{+3} + H_2O \leftrightarrow AmOH^{+2} + H^+$	-7.2
$Am^{+3} + 2H_2O \leftrightarrow Am(OH)_2{}^+ + 2H^+$	-15.1
$Am^{+3} + 3H_2O \leftrightarrow Am(OH)_3 + 3H^+$	-26.2
$Am^{+3} + CO_3^{-2} \leftrightarrow AmCO_3{}^+$	7.9
$Am^{+3} + 2CO_3^{-2} \leftrightarrow Am(CO_3)_2{}^-$	12.6
$Am^{+3} + 3CO_3^{-2} \leftrightarrow Am(CO_3)_3{}^{-3}$	14.6
$Am^{+3} + H^+ + CO_3^{-2} \leftrightarrow AmHCO_3{}^{+2}$	13.43
$Am^{+3} + Cl^- \leftrightarrow AmCl^{+2}$	0.24
$Am^{+3} + 2Cl^- \leftrightarrow AmCl_2{}^+$	-0.81

Note: The equations and thermodynamic constants are sourced from ThermoChimie database[1]

Table S2. Specific ion interaction theory (SIT) coefficients from ThermoChimie database [1].

Anion	Cation	SIT coefficients
Cl^-	Na^+	0.03
$ClO_4{}^-$	Na^+	0.01
OH^-	Na^+	0.04
$HCO_3{}^-$	Na^+	0.00
$CO_3{}^{-2}$	Na^+	-0.08
Cl^-	H^+	0.12
$ClO_4{}^-$	H^+	0.14
Cl^-	Am^{+3}	0.23
Cl^-	$AmOH^{+2}$	-0.04
Cl^-	$Am(OH)_2{}^+$	-0.29
Cl^-	$AmCO_3{}^+$	0.21
Cl^-	$AmHCO_3{}^{+2}$	-0.04*
Cl^-	$AmCl^{+2}$	0.191
Cl^-	$AmCl_2{}^+$	0.129
$ClO_4{}^-$	Am^{+3}	0.49
$ClO_4{}^-$	$AmOH^{+2}$	0.39
$ClO_4{}^-$	$Am(OH)_2{}^+$	0.17
$ClO_4{}^-$	$AmCO_3{}^+$	0.21
$ClO_4{}^-$	$AmHCO_3{}^{+2}$	0.39*
$ClO_4{}^-$	$AmCl^{+2}$	0.39
$ClO_4{}^-$	$AmCl_2{}^+$	0.17
$Am(CO_3)_2{}^-$	Na^+	0.06

$Am(CO_3)_3^{-3}$	Na^+	0.08
Cl^-	Eu^{+3}	0.23
Cl^-	$EuOH^{+2}$	-0.04
Cl^-	$Eu(OH)_2^+$	-0.29
Cl^-	$EuCO_3^+$	0.129
Cl^-	$EuHCO_3^{+2}$	-0.04#
Cl^-	$EuCl^{+2}$	0.191
Cl^-	$EuCl_2^+$	0.129
ClO_4^-	Eu^{+3}	0.49
ClO_4^-	$EuOH^{+2}$	0.39
ClO_4^-	$Eu(OH)_2^+$	0.17
ClO_4^-	$EuCO_3^+$	0.17
ClO_4^-	$EuHCO_3^{+2}$	0.39#
ClO_4^-	$EuCl^{+2}$	0.39
ClO_4^-	$EuCl_2^+$	0.17$
$Eu(CO_3)_2^-$	Na^+	-0.14
$Eu(CO_3)_3^{-3}$	Na^+	-0.23
$Eu(OH)_4^-$	Na^+	-0.14!

Note: *Adopted from $AmOH^{+2}$
#Adopted from $EuOH^{+2}$
$Adopted from $EuCO3^+$
!Adopted from $Eu(CO_3)_2^-$

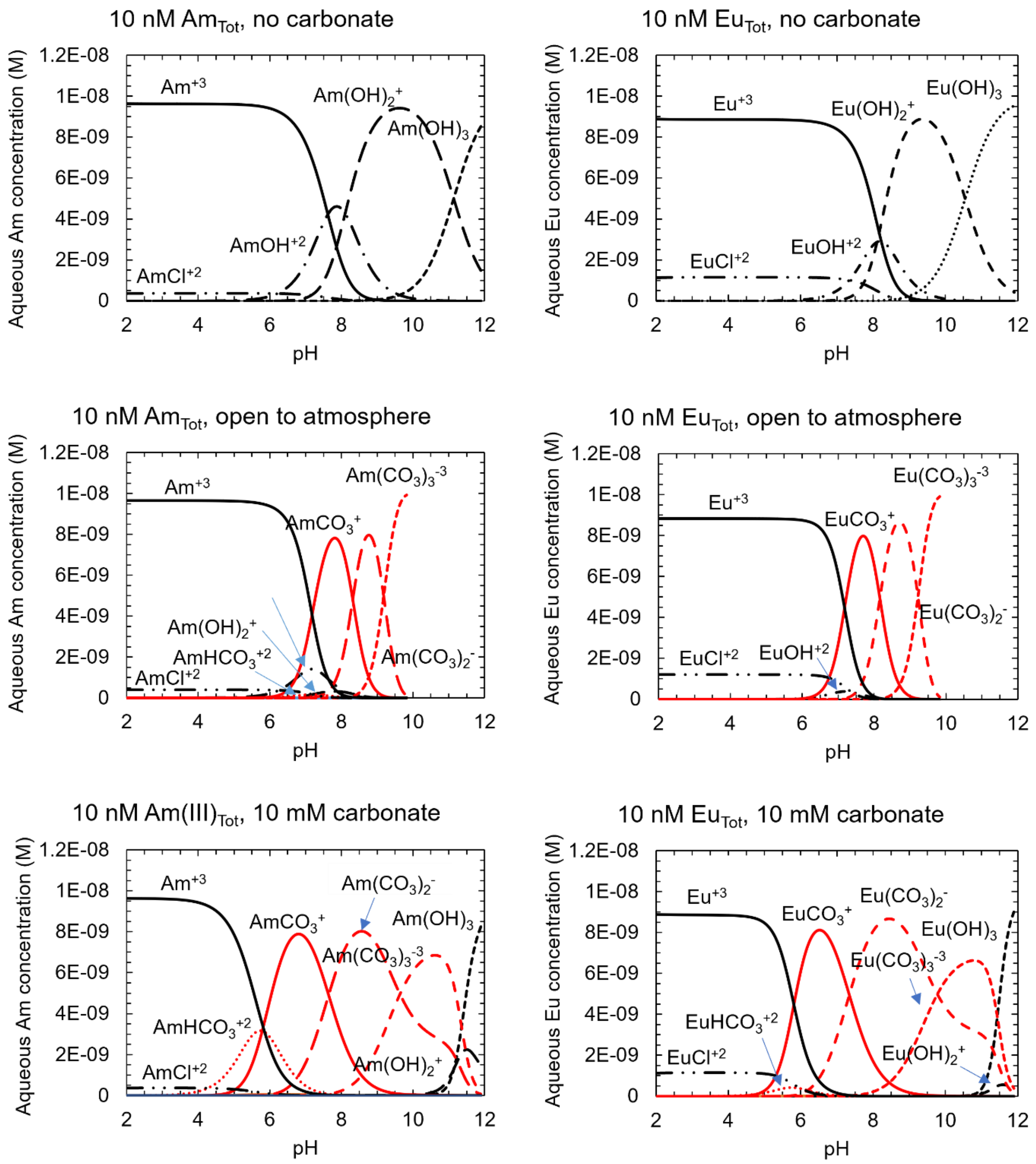

Figure S1. Aqueous speciation of Eu(III) and Am(III) as a function of pH and carbonate concentration. Equilibrium calculations were performed using PHREEQC by specific ion interaction theory (SIT). SIT parameters and log K values were sourced from ThermoChimie database[1] and are summarized in Tables S1 and S2.

Table S3. Experimental conditions for Eu(III)-corundum adsorption data.

Dataset ID	Eu(III) total	Sorbent Loading	Specific Surface Area	Background Electrolyte	Ionic Strength	CO_2(g) Partial Pressure	Source
	(M)	($g\cdot L^{-1}$)	($m^2\cdot g^{-1}$)		(M)	(log atm)	
**1a**	7.00×10^{-9}	5.00	0.023887	$NaClO_4$	0.01	-3.4	Norden et al. [2]
**1b**	7.00×10^{-9}	5.00	0.023887	$NaClO_4$	0.1	-3.4	
**2a**	6.60×10^{-9}	6.00	14.5	$NaClO_4$	0.1	-	Kupcik et al. [3]
**2b**	6.60×10^{-8}	6.00	14.5	$NaClO_4$	0.1	-	
**2c**	6.60×10^{-7}	6.00	14.5	$NaClO_4$	0.1	-	
**2d**	6.60×10^{-6}	6.00	14.5	$NaClO_4$	0.1	-	
**2e**	6.60×10^{-5}	6.00	14.5	$NaClO_4$	0.1	-	
**3a**	1.00×10^{-8}	0.50	4.8	NaCl	0.01	-3.4	Baumer et al. [4]
**3b**	1.00×10^{-8}	1.01	4.8	NaCl	0.01	-3.4	
**3c**	1.00×10^{-8}	6.15	4.8	NaCl	0.01	-3.4	
**3d**	1.00×10^{-5}	0.52	4.8	NaCl	0.01	-3.4	
**3e**	1.00×10^{-5}	1.04	4.8	NaCl	0.01	-3.4	
**3f**	1.00×10^{-5}	1.04	4.8	NaCl	0.1	-3.4	
**3g**	1.00×10^{-5}	3.13	4.8	NaCl	0.1	-3.4	

Table S4. Experimental conditions for Eu(III)-γ-alumina adsorption data.

Dataset ID	Eu(III) total (M)	Sorbent Loading ($g \cdot L^{-1}$)	Specific Surface Area ($m^2 \cdot g^{-1}$)	Background Electrolyte	Ionic Strength (M)	CO_2(g) Partial Pressure (log atm)	Source
**1a**	3.10×10^{-5}	3.60	119	$NaClO_4$	0.1	-3.4	Rabung et al. [5]
**1b**	2.10×10^{-5}	3.60	119	$NaClO_4$	0.1	-3.4	
**1c**	1.10×10^{-5}	3.60	119	$NaClO_4$	0.1	-3.4	
**1d**	5.20×10^{-6}	3.60	119	$NaClO_4$	0.1	-3.4	
**1e**	9.00×10^{-8}	3.60	119	$NaClO_4$	0.1	-3.4	
**2a**	1.00×10^{-3}	4.00	135.5	$NaNO_3$	0.1	-3.4	Morel et al. [6]
**3a**	1.00×10^{-7}	3.00	203	$NaClO_4$	0.1	-3.4	Kumar et al. [7]
**3b**	1.00×10^{-6}	3.00	203	$NaClO_4$	0.1	-3.4	
**3c**	1.00×10^{-5}	3.00	203	$NaClO_4$	0.1	-3.4	
**3d**	1.00×10^{-4}	3.00	203	$NaClO_4$	0.1	-3.4	
**4a**	1.00×10^{-8}	0.04	58.1	NaCl	0.01	-3.4	Baumer et al. [4]
**4b**	1.01×10^{-8}	0.09	58.1	NaCl	0.01	-3.4	
**4c**	1.00×10^{-8}	0.51	58.1	NaCl	0.01	-3.4	
**4d**	1.00×10^{-5}	0.04	58.1	NaCl	0.01	-3.4	
**4e**	1.00×10^{-5}	0.09	58.1	NaCl	0.01	-3.4	

Table S5. Experimental conditions for Eu(III)-gibbsite adsorption data sourced from Baumer et al. [4].

Dataset ID	Eu(III) total (M)	Sorbent Loading ($g \cdot L^{-1}$)	Specific Surface Area ($m^2 \cdot g^{-1}$)	Background Electrolyte	Ionic Strength (M)	CO_2(g) Partial Pressure (log atm)
**1a**	1.16×10^{-8}	0.039746	64.47	NaCl	0.01	-3.4
**1b**	1.10×10^{-8}	0.079663	64.47	NaCl	0.01	-3.4
**1c**	1.09×10^{-8}	0.540389	64.47	NaCl	0.01	-3.4
**1d**	9.88×10^{-6}	0.039436	64.47	NaCl	0.01	-3.4
**1e**	9.62×10^{-6}	0.076807	64.47	NaCl	0.01	-3.4

Table S6. Experimental conditions for Am(III)-corundum adsorption data.

Dataset ID	Am(III) total (M)	Sorbent Loading ($g \cdot L^{-1}$)	Specific Surface Area ($m^2 \cdot g^{-1}$)	Background Electrolyte	Ionic Strength (M)	CO_2(g) Partial Pressure (log atm)	Source
**1a**	2.9×10^{-7}	10	0.01438	$NaClO_4$	0.1	-3.4	Allard et al.[8]
**1b**	2.3×10^{-9}	10	0.01438	$NaClO_4$	0.1	-3.4	
**2a**	1.0×10^{-8}	10	0.07	$NaClO_4$	0.01	-3.4	Moulin et al.[9]
**2b**	1.0×10^{-8}	10	0.07	$NaClO_4$	0.1	-3.4	

Table S7. Experimental conditions for Am(III)-γ-alumina adsorption data.

Dataset ID	Am(III) total (M)	Sorbent Loading ($g \cdot L^{-1}$)	Specific Surface Area ($m^2 \cdot g^{-1}$)	Background Electrolyte	Ionic Strength (M)	CO_2(g) Partial Pressure (log atm)	Source
**1a**	5.0×10^{-10}	0.01	130	$NaClO_4$	0.1	-3.4	Righetto et al., 1988[10]
**1b**	5.0×10^{-10}	0.2	130	$NaClO_4$	0.1	-3.4	
**1c**	5.0×10^{-10}	0.2	130	$NaClO_4$	0.01	-3.4	
**2a**	5.0×10^{-10}	0.2	130	$NaClO_4$	0.1	-3.4	Righetto et al., 1991[11]

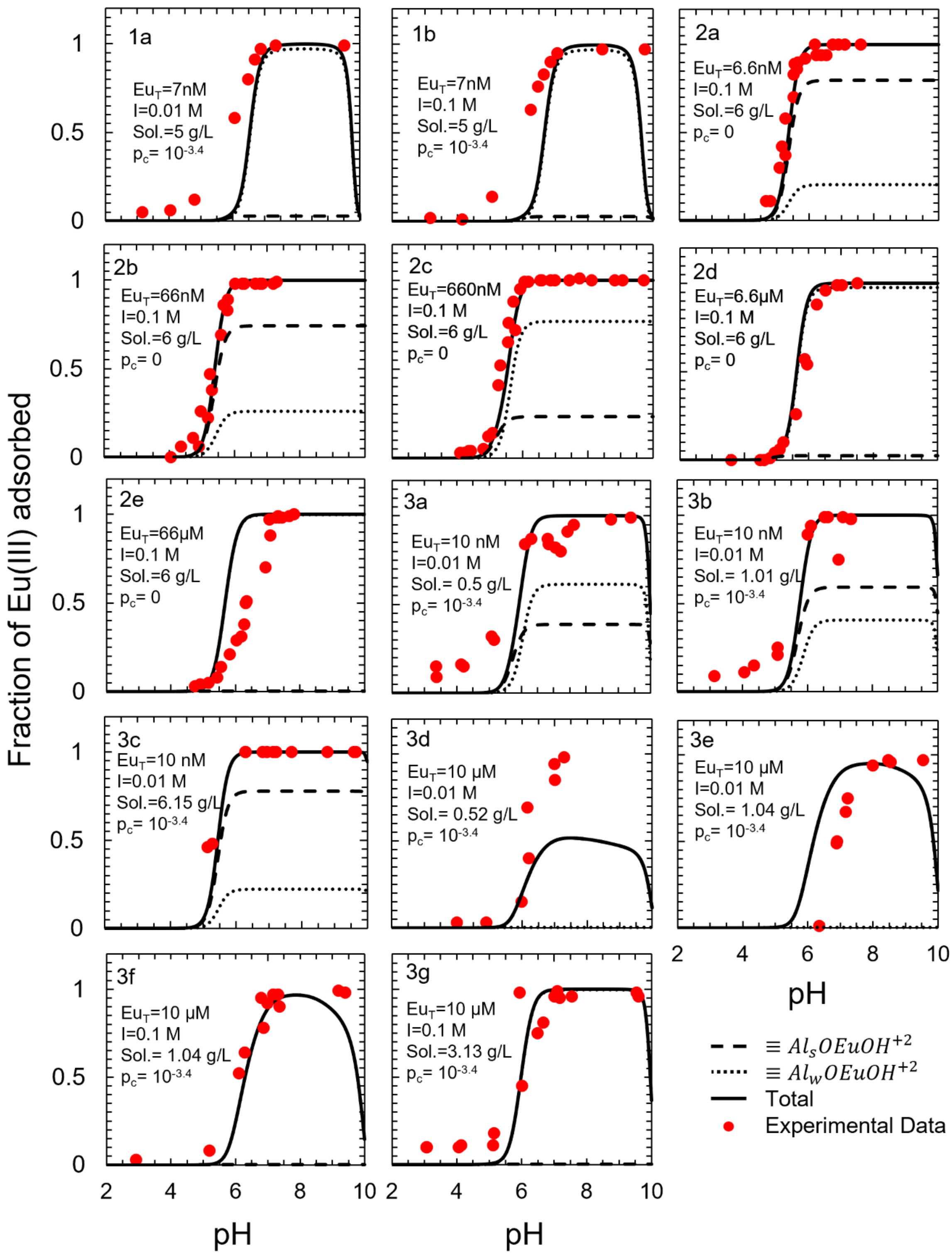

Figure S2. Surface complexation speciation of the best fit DDL model describing Eu(III) adsorption to corundum as a function of total europium concentration (Eu_T), ionic strength (I), sorbent solid concentration (Sol.), and partial pressure of $CO_{2(g)}$ (p_c). Experimental data were derived from (1) Norden et al. [2] (2) Kupcik et al. [3] and (3) Baumer et al. [4].

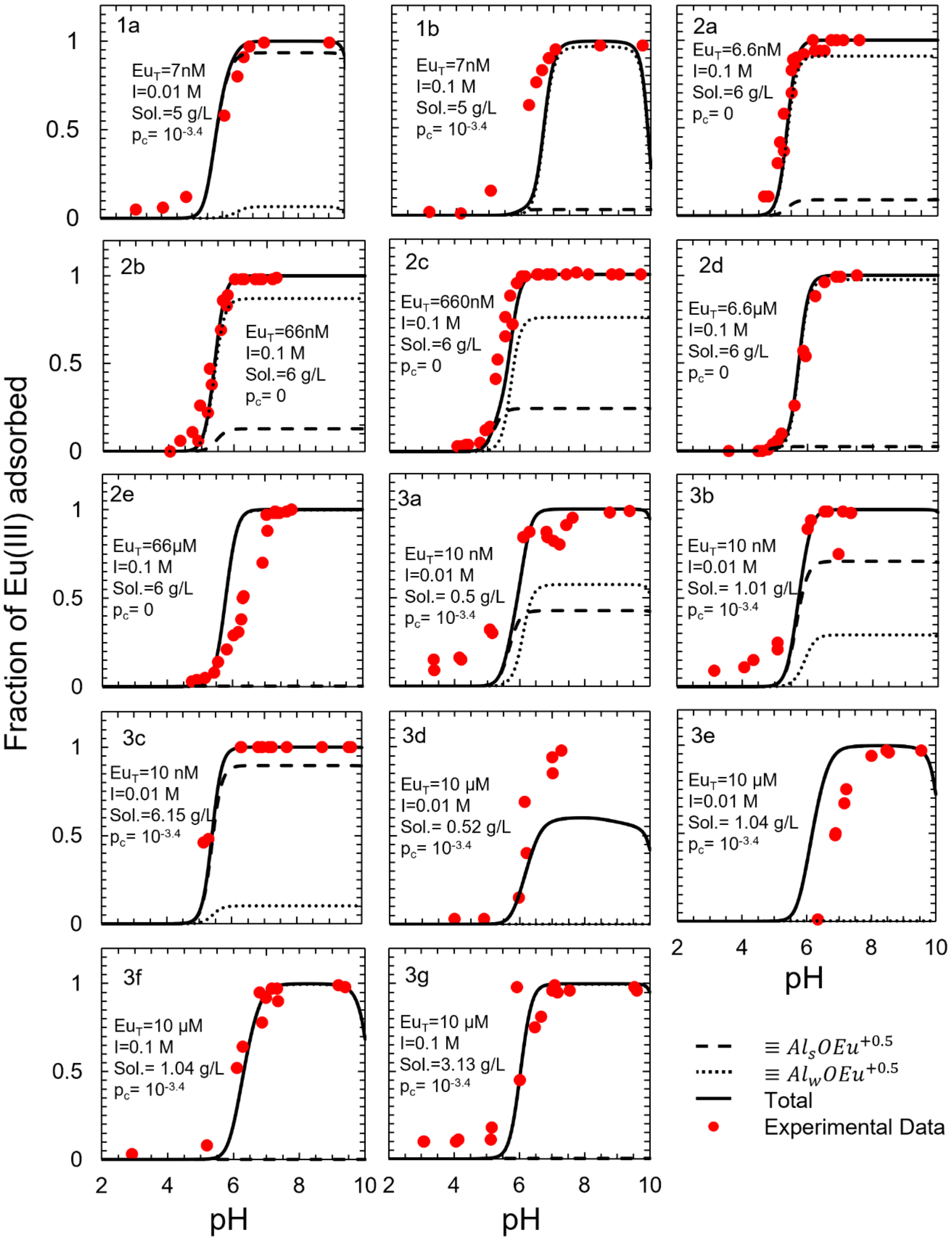

Figure S3. Surface complexation speciation of the best fit CD-MUSIC model describing Eu(III) adsorption to corundum as a function of total europium concentration (Eu_T), ionic strength (I), sorbent solid concentration (Sol.), and partial pressure of $CO_{2(g)}$ (p_c). Experimental data were derived from (1) Norden et al. [2] (2) Kupcik et al. [3] and (3) Baumer et al. [4].

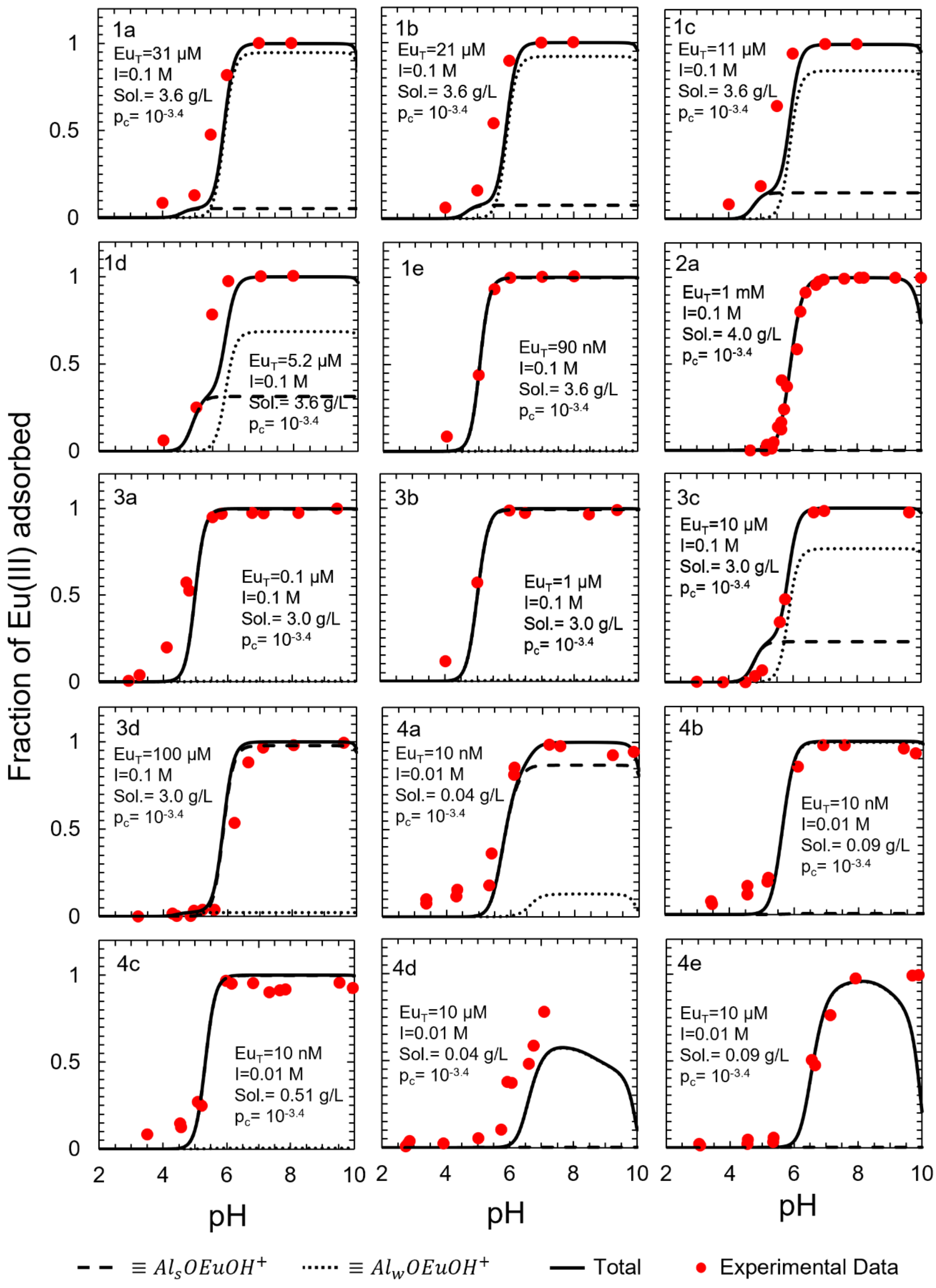

Figure S4. Surface complexation speciation of the best-fit DDL model describing Eu(III) adsorption to gamma-alumina as a function of total europium concentration (Eu_T), ionic strength (I), sorbent solid

concentration (Sol.), and partial pressure of $CO_{2(g)}$ (p_c). Experimental data were derived from (1) Rabung et al. [5] (2) Morel et al. [6] (3) Kumar et al. [12] and (4) Baumer et al. [4]

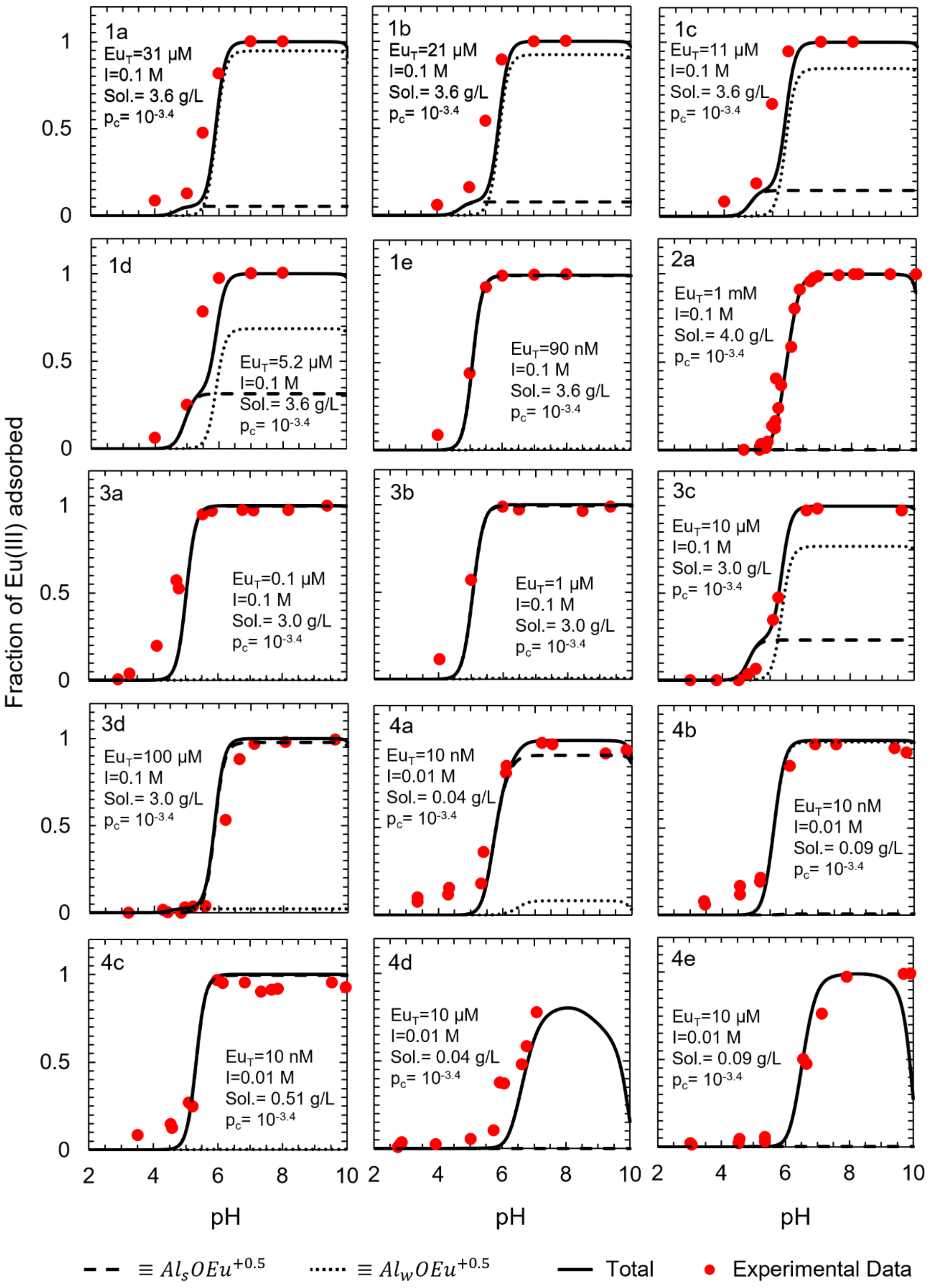

Figure S5. Surface complexation speciation of the best-fit DDL model describing Eu(III) adsorption to

gamma-alumina as a function of total europium concentration (Eu_T), ionic strength (I), sorbent solid concentration (Sol.), and partial pressure of $CO_{2(g)}$ (p_c). Experimental data were derived from (1) Rabung et al. [5] (2) Morel et al. [6] (3) Kumar et al. [12] and (4) Baumer et al. [4]

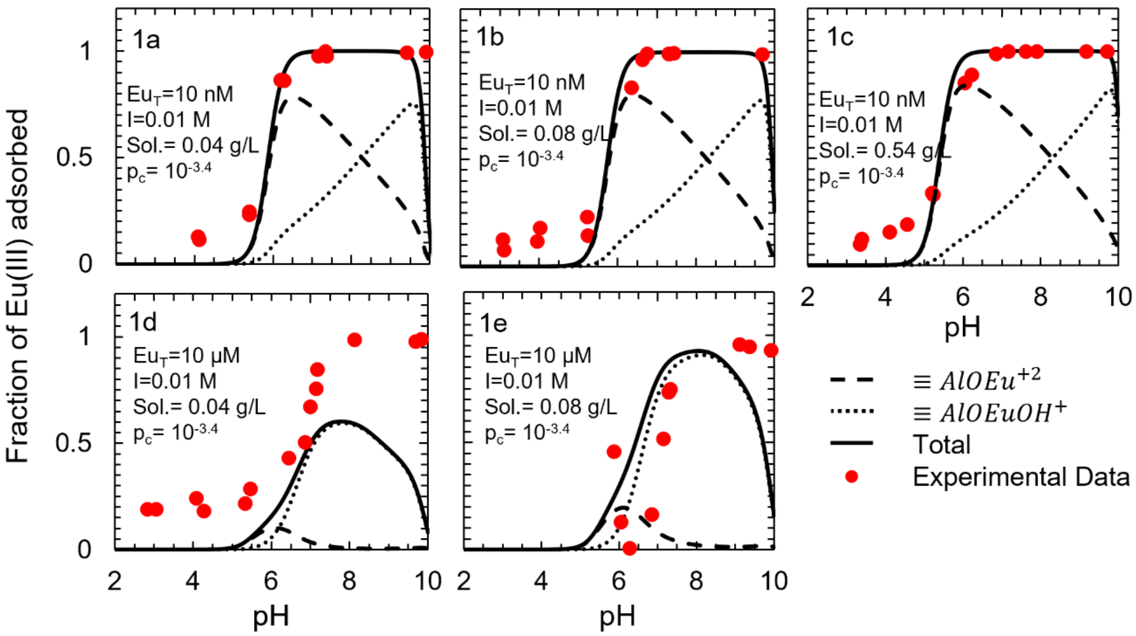

Figure S6. Surface complexation models describing Eu(III) adsorption to gibbsite as a function of total europium concentration (Eu_T), ionic strength (I), sorbent solid concentration (Sol.), and partial pressure of $CO_{2(g)}$ (p_c). Experimental data were derived from Baumer et al. [4]

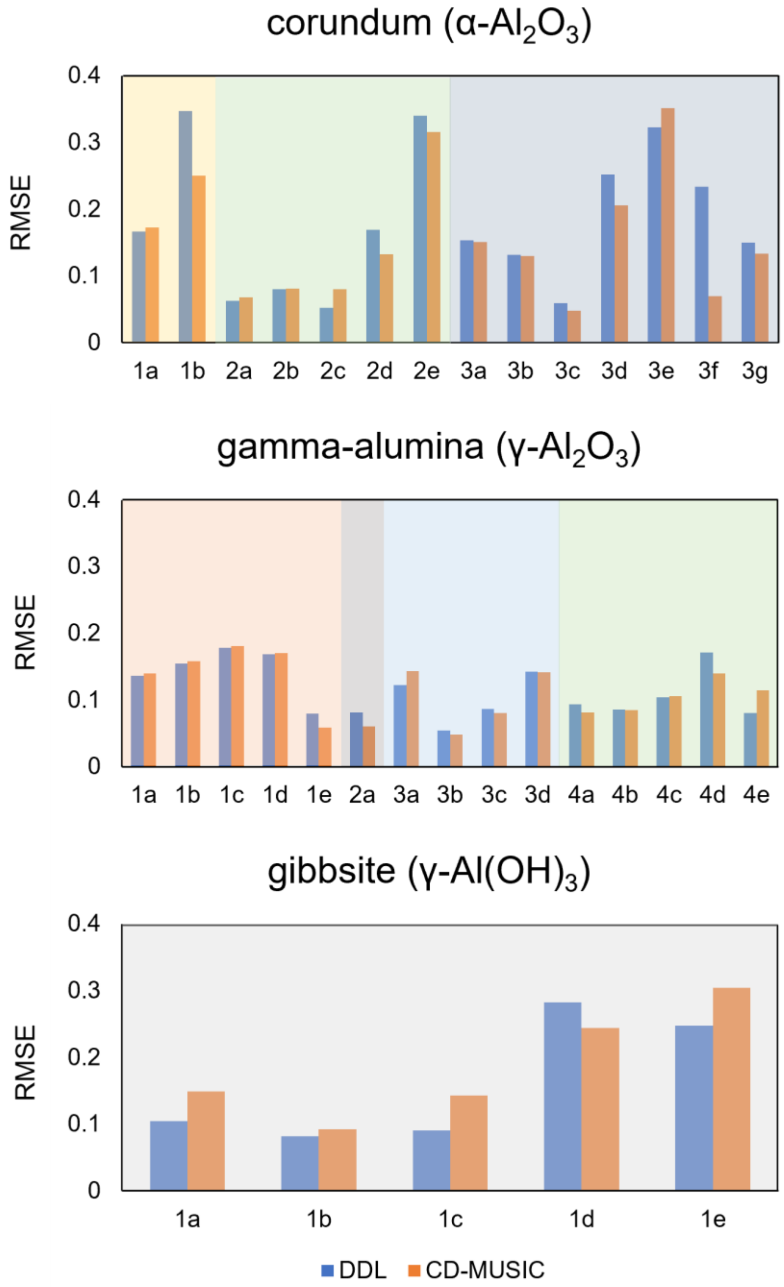

Figure S7. The root mean square of error (RMSE) for the DDL and the CD-MUSIC models developed in this work for different Eu(III)-aluminum (hydr)oxide adsorption datasets. Refer Table S3 (corundum), Table S4 (γ-alumina), and Table S5 (gibbsite) for the experimental conditions corresponding to each dataset.

Table S8. Surface complexation models for Eu(III) adsorption on γ-alumina.

Source literature	Site conc. (mol·g^{-1})	Model type	Capacitance (F·m^{-2})	log $K_{a1}$$^{@}$	log $K_{a2}$$^{!}$	log K_1*	log $K_2$$^{\#}$
Rabung et al. [5]	0.000199	CCM	0.8	6.87	-10.40	2.58$^{\$}$	--
Kumar et al. [7]	0.000136	CCM	1.2	7.2	-9.1	2.21	-4.98
Morel et al. [6]	0.000385	DDL	N/A	7.9	-9.22	-1.2	--

Note: $^{\$}$for strong sites which are 2% of the total site concentration.
CCM=Constant Capacitance Model
DDL=Diffuse Double Layer Model
$^{@}\equiv AlOH + H^{+} \leftrightarrow \equiv AlOH_2{}^{+}$
$^{!}\equiv AlOH \leftrightarrow \equiv AlO^{-} + H^{+}$
$^{*}\equiv AlOH + Am^{+3} \leftrightarrow \equiv AlOAm^{+2} + H^{+}$
$^{\#}\equiv AlOH + Am^{+3} \leftrightarrow (\equiv AlO)_2Am^{+} + 2H^{+}$

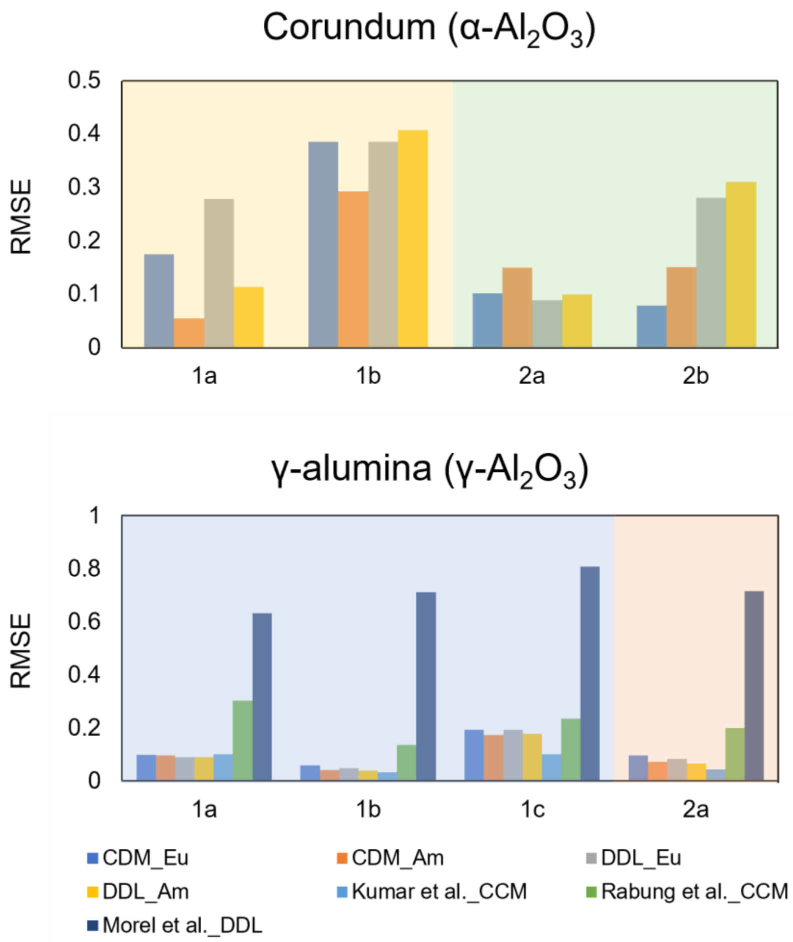

Figure S8. The root mean square of error (RMSE) for the DDL and the CD-MUSIC models developed in this work for corundum and γ-alumina, and the previous models sourced from literature for Am(III) adsorption data. Refer Table S6 (corundum), and Table S7 (γ-alumina) for the experimental conditions corresponding to each dataset.

Note: CDM_Eu=CD-MUSIC model developed by employing europium sorption data
CDM_Am=CD-MUSIC model developed by employing americium sorption data
DDL_Eu=DDL model developed by employing europium sorption data
DDL_Am=DDL model developed by employing americium sorption data
Kumar et al._CCM[7], Rabung et al._CCM[5] and Morel et al._DDL[6] are previous models sourced from literature.

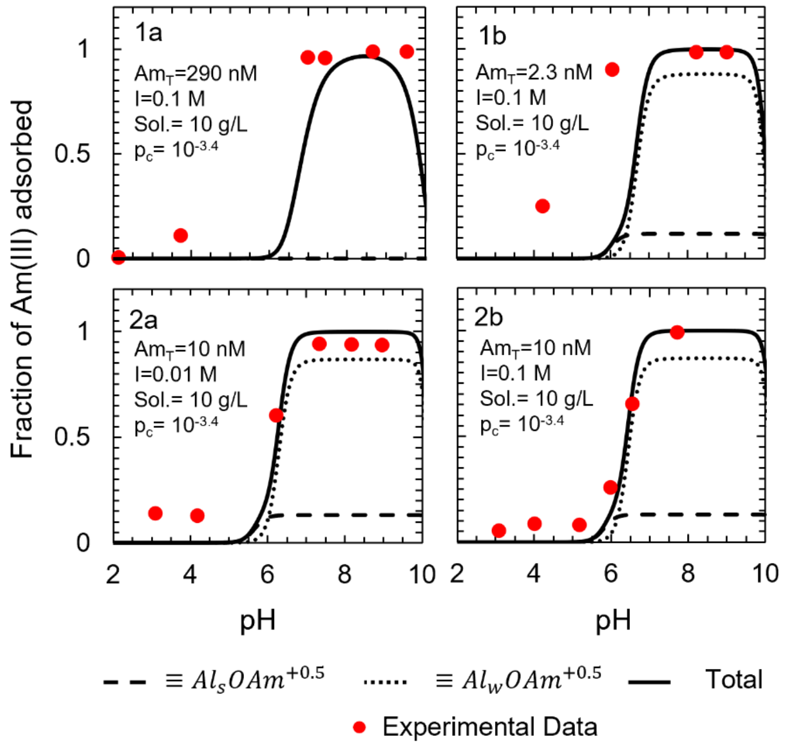

Figure S9. Surface speciation of the CD-MUSIC model developed by employing Eu(III) adsorption data describing Am(III) adsorption to corundum as a function of total americium concentration (Am_T), ionic strength (I), sorbent solid concentration (Sol.), and partial pressure of $CO_{2(g)}$ (p_c). Experimental data were derived from (1) Allard et al.[8] and (2) Moulin et al.[9] and full experimental conditions are summarized in Table S6.

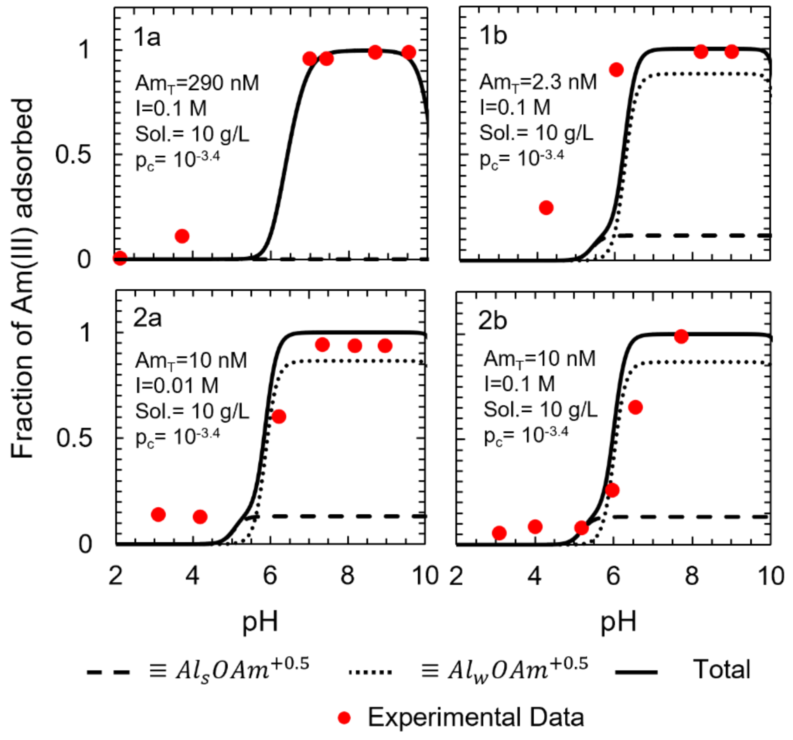

Figure S10. Surface speciation of the CD-MUSIC model developed by employing Am(III) adsorption data describing Am(III) adsorption to corundum as a function of total americium concentration (Am_T), ionic strength (I), sorbent solid concentration (Sol.), and partial pressure of $CO_{2(g)}$ (p_c). Experimental data were derived from (1) Allard et al.[8] and (2) Moulin et al.[9] and full experimental conditions are summarized in Table S6.

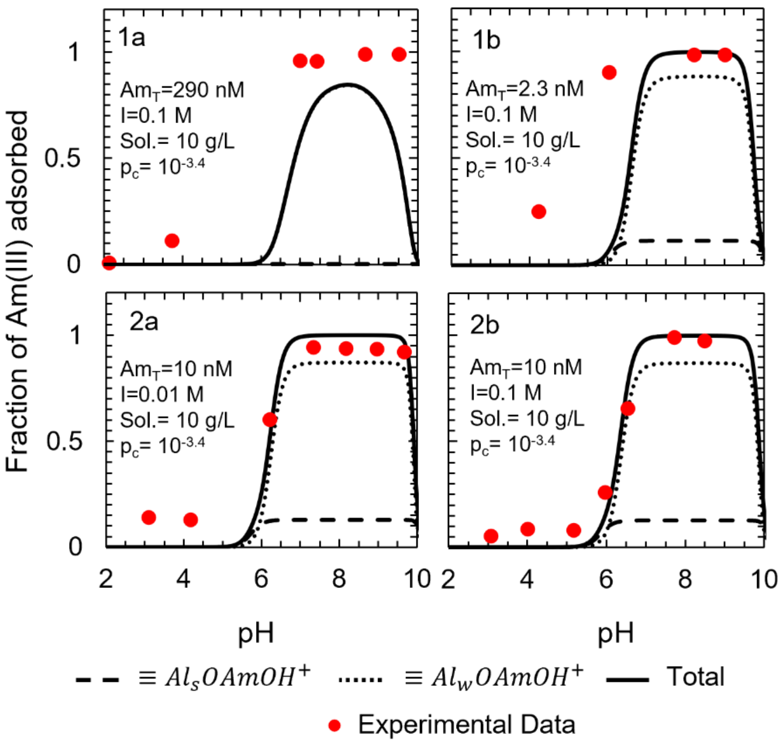

Figure S11. Surface speciation of the DDL model developed by employing Eu(III) adsorption data describing Am(III) adsorption to corundum as a function of total americium concentration (Am_T), ionic strength (I), sorbent solid concentration (Sol.), and partial pressure of $CO_{2(g)}$ (p_c). Experimental data were derived from (1) Allard et al.[8] and (2) Moulin et al.[9] and full experimental conditions are summarized in Table S6.

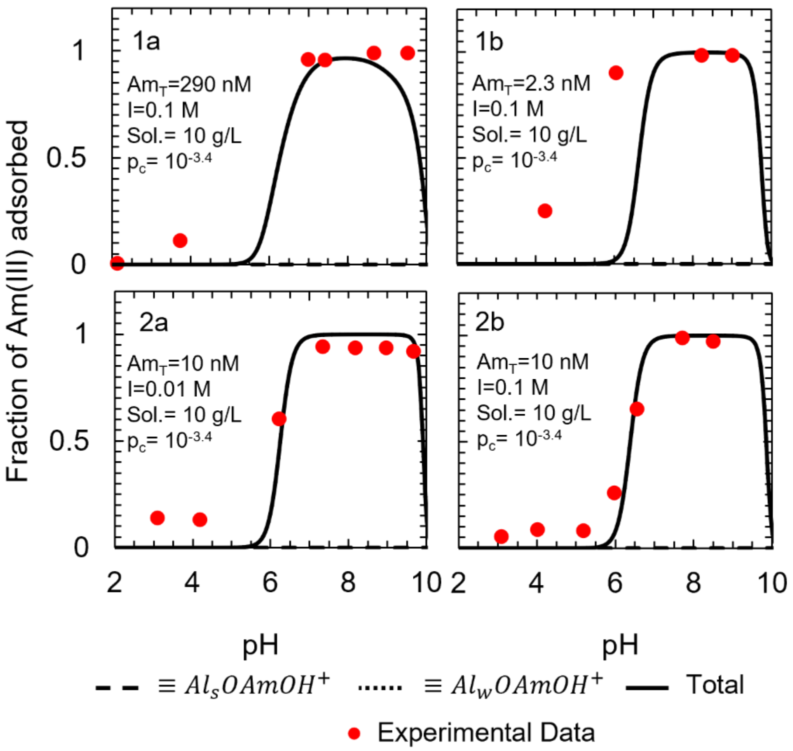

Figure S12. Surface speciation of the DDL model developed by employing Am(III) adsorption data describing Am(III) adsorption to corundum as a function of total americium concentration (Am_T), ionic strength (I), sorbent solid concentration (Sol.), and partial pressure of $CO_{2(g)}$ (p_c). Experimental data were derived from (1) Allard et al.[8] and (2) Moulin et al.[9] and full experimental conditions are summarized in Table S6.

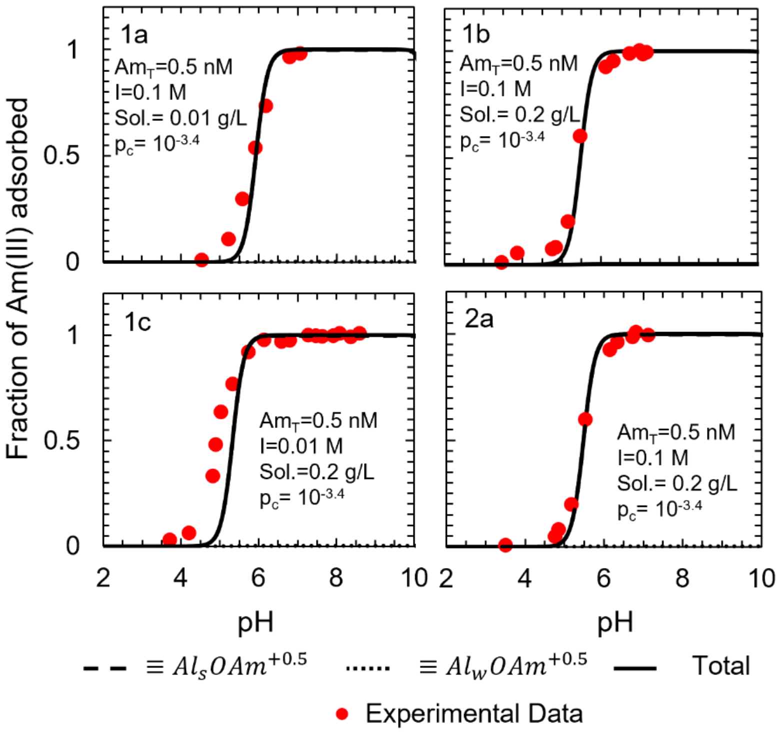

Figure S13. Surface speciation of the CD-MUSIC model developed by employing Eu(III) adsorption data describing Am(III) adsorption to corundum as a function of total americium concentration (Am_T), ionic strength (I), sorbent solid concentration (Sol.), and partial pressure of $CO_{2(g)}$ (p_c). Experimental conditions from (1) Righetto et al., 1988[10] and (2) Righetto et al., 1991[11] and full experimental conditions are summarized in Table S7.

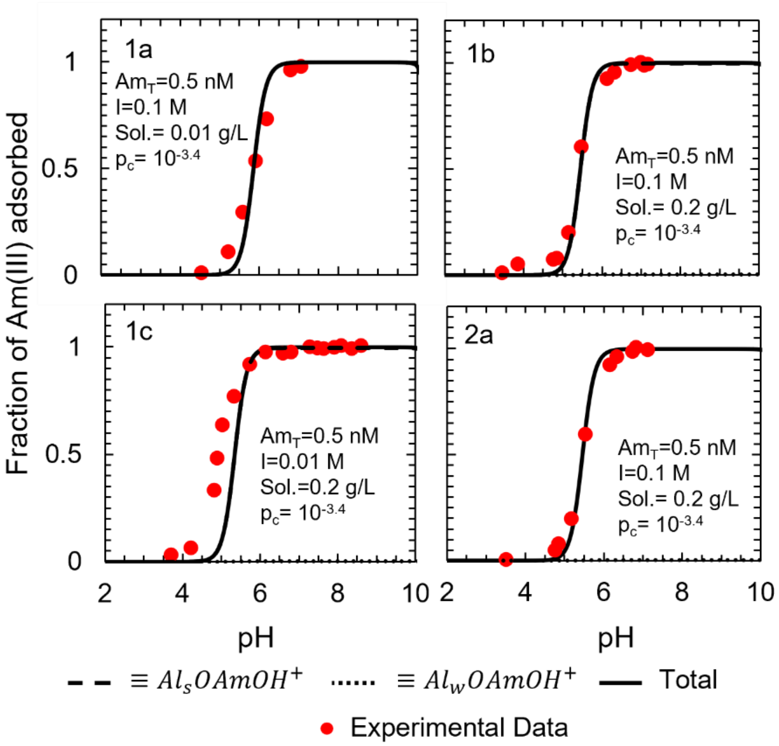

Figure S14. Surface speciation of the DDL model developed by employing Eu(III) adsorption data describing Am(III) adsorption to corundum as a function of total americium concentration (Am_T), ionic strength (I), sorbent solid concentration (Sol.), and partial pressure of $CO_{2(g)}$ (p_c). Experimental conditions from (1) Righetto et al., 1988[10] and (2) Righetto et al., 1991[11] and full experimental conditions are summarized in Table S7.

References

1. *ThermoChimie Thermodynamic Database*. 2022 [cited 2022 11/10/2022]; Available from: http://www.thermochimie-tdb.com/.
2. Norden, M., J. Ephraim, and B. Allard, *The influence of a fulvic acid on the adsorption of europium and strontium by alumina and quartz: effects of pH and ionic strength.* Radiochimica Acta, 1994. **65**(4): p. 265-270.
3. Kupcik, T., et al., *Macroscopic and spectroscopic investigations on Eu (III) and Cm (III) sorption onto bayerite (β-Al (OH) 3) and corundum (α-Al2O3).* Journal of colloid and interface science, 2016. **461**: p. 215-224.
4. Baumer, T., P. Kay, and A.E. Hixon, *Comparison of europium and neptunium adsorption to aluminum (hydr) oxide minerals.* Chemical Geology, 2017. **464**: p. 84-90.
5. Rabung, T., et al., *Sorption of Am (III) and Eu (III) onto γ-alumina: experiment and modelling.* Radiochimica Acta, 2000. **88**(9-11): p. 711-716.
6. Morel, J.-P., et al., *Effect of temperature on the sorption of europium on alumina: Microcalorimetry and batch experiments.* Journal of colloid and interface science, 2012. **376**(1): p. 196-201.
7. Kumar, S., S. Godbole, and B. Tomar, *Speciation of Am (III)/Eu (III) sorbed on γ-alumina: effect of metal ion concentration.* Radiochimica Acta, 2013. **101**(2): p. 73-80.
8. Allard, B., et al., *Sorption of actinides in well-defined oxidation states on geologic media.* MRS Online Proceedings Library (OPL), 1981. **11**.
9. Moulin, V., D. Stammose, and G. Ouzounian, *Actinide sorption at oxide-water interfaces: application to α alumina and amorphous silica.* Applied geochemistry, 1992. **7**: p. 163-166.
10. RIGHETTO, L., et al., *Surface interactions of actinides with alumina colloids.* Radiochimica Acta, 1988. **44**(1): p. 73-76.
11. Righetto, L., et al., *Competitive actinide interactions in colloidal humic acid-mineral oxide systems.* Environmental science & technology, 1991. **25**(11): p. 1913-1919.
12. *SOURCE CLAY PHYSICAL/CHEMICAL DATA.* 05/16/2020]; Available from: http://www.clays.org/sourceclays_data.html
